# Supplementary material for: Phylogenomic and Evolutionary Insights into Lipoprotein Lipase (LPL) Genes in Tambaqui: Gene Duplication, Tissue-Specific Expression and Physiological Implications
Source: Genes (Basel). 2025 Apr 30;16(5):548. doi: 10.3390/genes16050548 (PMC12111056; doi:10.3390/genes16050548)
Supplement: Supplementary file 1 [file genes-16-00548-s001.zip › genes-3572228-supplementary.pdf]

Supplementary information

Table S1. Primer sequences used for quantitative real-time PCR (qPCR). The table includes forward and reverse primer sequences for target genes.

Primer ID	Primer sequence 5’-3’	Length	Tm (°C)	Amplicon
cm.lpl1a forward	GAGAGACAGTCCAAAGTGATC	21	60	174
cm.lpl1a reverse	CTATTCTGTGCTCTTCTTGAAG	22	59	
cm.lpl1bforward	GAGAGACACAAACCAAGGTG	20	60	237
cm.lpl1b reverse	CTGTGGACTCTTCTTTACTGG	21	60	
cm.lpl2a forward	CCTCCAACTTAAAGGGGATG	20	60	188
cm.lpl2a reverse	GTTCAGAACCAGTAGTATCTCC	22	59	

Figure S1. Infographic of lipoprotein lipase protein sequences from different vertebrates. The figure shows the protein sequences along with their corresponding taxonomic groups, GenBank sequence IDs, and genomic locations.

Classification		Species	LPL1	*lpl1* Location			LPL2	*lpl2* Location	
Chondrichthyes		*Callorhinchus milii*	XP_007899209.1	NW_024704762.1			XP_007899210.1	NW_024704762.1	
Sarcopterygii	Coelacanth	*Latimeria chalumnae*	XP_005989949.1	Chr. 21			XP_064424649.1	Chr. 21	
	Mammals	*Homo sapiens*	NP_000228.1	Chr. 8					
	Birds	*Gallus gallus*	XP_015135900.1	Chr. Z					
	Amphibian	*Xenopus tropicalis*	XP_002934038.1	Chr. 1					
	Reptiles	*Podacis muralis*	XP_028568270.1	Chr. 17					
Actinopterygii	Non-teleost	*Erpetoichthys calabaricus*	XP_028672356.1	Chr. 12			XP_028672354.1	Chr. 12	
		Lepisosteus oculatus	XP_006640052.1	Chr. 29			XP_006640053.2	Chr. 29	
Teleost Whole genome duplication (3R)			Lpl1a	*lpl1a* Location	Lpl1b	*lpl1b* Location	Lpl2a	*lpl2a* Location	Lpl2b
Actinopterygii	Early branching teleost	*Anguilla anguilla*	XP_035272161.1	Chr. 4	XP_035276326.1<br>XP_035276363.1	Chr. 6<br>Chr. 6	XP_035270726.1	Chr. 4	
		Megalops cyprinoides	XP_036409940.1	Chr. 20	XP_036376712.1	Chr. 2	XP_036374600.1	Chr. 20	
		Scleropages formosus	XP_018583848.2	Chr. 9			XP_018583841.2	Chr. 9	
		Paramormyrops kingsleyae	XP_023699829.1	Chr. 15	XP_072560370.1	Chr. 21	XP_023699905.1	Chr. 15	XP_023674674.1<br>(Chr. 21)
	Otocephala	*Clupea harengus*	XP_012683808.2	Chr. 10	XP_042561452.1	IW_024879925.	XP_012683807.1	Chr. 10	
		Chanos chanos	XP_030640550.1	Chr. 9	XP_030630539.1	Chr. 5	XP_030639366.1	Chr. 9	
		Danio rerio	NP_571202.1	Chr. 22	XP_005174466.1	Chr. 2	XP_002666287.3	Chr. 22	
		Cyprinus carpio	XP_018919916.2<br>XP_042568359.1<br>XP_042568381.1	Chr. B22<br>Chr. A22<br>Chr. A22	XP_042575005.1	Chr. B2	XP_042604741.1	Chr. B22	
		Pangasianodon hypophthalmus	XP_026786482.1	Chr. 11	XP_034153683.1	Chr. 21	XP_026786600.2	Chr. 11	
		Ictalurus punctatus	NP_001316205.1	Chr. 10	XP_017351928.1	Chr. 20			
		Electrophorus electricus	XP_026861273.2	Chr. 26	XP_026874726.2	Chr. 7	XP_026861314.2	Chr. 26	
		Astyanax mexicanus	XP_007240188.3	Chr. 16	XP_049334816.1	Chr. 4	XP_007240187.1	Chr. 16	
		Pygocentrus nattereri	XP_017548830.1	Chr. 15	XP_017552653.2	Chr. 19	XP_017548831.1	Chr. 15	
		Colossoma macropomum	XP_036442904.1	Chr. LG19	XP_036412281.1	Chr. LG23	XP_036442371.1	Chr. LG19	
	Euteleostei	*Esox lucius*	XP_010869814.1	Chr. 8	XP_010889273.1	Chr. 3	XP_019904837.1	Chr. 8	
		Salmo salar	XP_014004153.1<br>XP_014071701.1	Chr. ssa16<br>Chr. ssa10	XP_013996657.1	Chr. ssa14	XP_045553204.1<br>XP_014071700.2	Chr. ssa16<br>Chr. ssa10	
		Oncorhynchus mykiss	XP_021435556.2<br>NP_001118076.1	Chr. 1<br>Chr. 5	XP_036842445.1	Chr. 8	XP_021435376.2<br>XP_021459327.2	Chr. 1<br>Chr. 5	
		Gadus morhua	XP_030228467.1	Chr. 12	XP_030219625.1	Chr. 8	XP_030228828.1	Chr. 12	
		Myripristis murdjan	XP_029905863.1	Chr. 4	XP_029931044.1	Chr. 17	XP_029905846.1	Chr. 4	
		Oreochormis niloticus	NP_001266682.1	Chr. LG23			XP_003439890.1	Chr. LG23	
		Oryzias latipes	NP_001295924.1	Chr. 4			XP_004068405.1	Chr. 4	
		Perca fluviatilis	XP_039666916.1	Chr. 9			XP_039667734.1	Chr. 9	
		Gasterosteus aculeatus	XP_040040562.1	Chr. 8			XP_040040767.1	Chr. 8	
		Dicentrarchus labrax	XP_051233158.1	NW_026136718.1			XP_051233284.1	NW_026136718.1	
		Takifugu rubripes	XP_003974174.2	Chr. 20			NP_001292532.1	Chr. 20	
		Scophthalmus maximus	XP_035504156.1	Chr. 13			XP_035504157.2	Chr. 13	
		Cynoglossus semilaevis	NP_001306750.1	Chr. 2			XP_008333524.1	Chr. 2	

Table S2. Physicochemical properties of tambaqui Lpl1a and Lpl1b, along with homologous proteins from other vertebrates (LPL), computed using the Expasy ProtParam server. The table includes taxonomic classification (Class: Chondrichthyes, Sarcopterygii, and Actinopterygii, including infraclasses), species, molecular weight (Da), isoelectric point (pI), instability index, and protein stability classification (stable/unstable).

Taxonomic classification	Species	Molecular Weight	pI	Instability Index	Protein stability classification
Condrichthyes LPL1	*C. milii*	55712.22	8.55	34.96	stable
Sarcopterygii LPL1/LPL	*L. chalumnae*	56016.80	8.49	38.73	stable
	H. sapiens	53162.50	8.37	42.35	unstable
Non-teleost LPL1	*E. calabaricus*	56924.84	8.42	40.82	unstable
	L. oculatus	56977.98	8.78	44.05	unstable
Early branching teleost Lpl1a	*P. kingslayae*	58049.08	8.64	46.88	unstable
Otocephala Lpl1a	*C. harengus*	57114.01	7.62	38.44	stable
	C. chanos	58180.47	8.22	41.89	unstable
	D. rerio	58291.64	8.25	41.68	unstable
	P. hypophthalmus	58079.71	8.74	41.50	unstable
	I. punctatus	58042.39	8.56	41.39	unstable
	E. electricus	57477.55	8.44	38.92	stable
	P. nattereri	57711.04	8.41	40.60	unstable
	C. macropomum	57713.03	8.23	40.82	unstable
	A. mexicanus	57745.25	7.58	43.72	unstable
Euteleostei Lpl1a	*Esox lucius*	57260.47	7.29	32.60	stable
	O. niloticus	58081.11	8.54	32.39	stable
	Oryzias latipes	57979.89	7.26	37.52	stable
	*G. morhu*a	57874.89	8.30	41.67	unstable
	M. murdjan	57140.01	6.58	35.75	stable
Early branching teleost Lpl1b	*P. kingslayae*	56548.74	8.65	43.51	unstable
Otocephala Lpl1b	*C. harengus*	56230.13	6.77	35.90	stable
	C. chanos	57295.43	8.40	31.13	stable
	D. rerio	60073.11	7.01	37.11	stable
	P. hypophthalmus	59042.24	8.69	29.75	stable
	I. punctatus	58828.06	8.25	29.55	stable
	E. electricus	56816.91	8.68	39.52	stable
	P. nattereri	59218.50	7.27	35.86	stable
	C. macropmum	60016.45	8.22	32.13	stable
	A. mexicanus	59499.64	7.31	34.58	stable
Euteleostei Lpl1b	*G. morhua*	54057.57	8.59	34.57	stable
	M. murdjan	56491.40	8.59	46.24	unstable

Table S3. Physicochemical properties of tambaqui Lpl2a, along with homologous proteins from other vertebrates (LPL2/Lpl2b), computed using the Expasy ProtParam server. The table includes taxonomic classification (Class: Chondrichthyes, Sarcopterygii, and Actinopterygii, including infraclasses), species, molecular weight (Da), isoelectric point (pI), instability index, and protein stability classification (stable/unstable).

Taxonomic classification	Species	Molecular Weight	pI	Instability Index	Protein stability classification
Condrichthyes LPL2	*C. milii*	57704.06	8.23	38.64	stable
Sarcopterygii LPL2	*L. chalumnae*	56184.27	7.39	42.49	unstable
Non teleost LPL2	*E. calabaricus*	56825.63	6.62	37.33	stable
	L. oculatus	55923.69	9.01	40.62	unstable
Early branching teleost Lpl2a	*P. kingslayae*	56309.16	8.36	33.44	stable
Otocephala Lpl2a	*C. harengus*	56853.90	7.95	37.84	stable
	C. chanos	56229.07	7.24	37.52	stable
	D. rerio	55794.86	6.81	35.10	stable
	P. hypophthalmus	55444.46	8.47	36.04	stable
	E. electricus	60203.09	9.17	38.59	stable
	P. nattereri	56275.12	8.37	32.04	stable
	C. macropomum	56522.42	8.51	32.36	stable
	A. mexicanus	57967.17	7.05	33.36	stable
Euteleostei Lpl2a	*E. lucius*	57196.13	6.78	35.06	stable
	O. niloticus	57471.63	8.80	30.74	stable
	O. latipes	57139.96	6.83	38.16	stable
	*G. morhu*a	56614.48	7.27	33.18	stable
	M. murdjan	57684.65	6.79	28.74	stable
Early branching teleost Lpl2b	*P. kingslayae*	57986.82	7.07	42.03	unstable

Figure S2. CLUSTAL multiple sequence alignment of lipoprotein lipase from tambaqui with vertebrate sequences. Expanded alignment of the deduced amino acid sequences of the lipoprotein lipase a and b gene from tambaqui with 32 vertebrate sequences. * indicates identical amino acid residues among multiple sequences. Gaps introduced to optimize alignment are represented by (-). Highlighted colors indicate domains previously described in human LPL. Alignment was performed using MUSCLE (3.8).

```
XP_007899209.1_Cmilii_LPL               ---MTG-------AVWIIC-LLSLVSFS-NTSAT---------DLQSDS----------D
XP_030219625.1_Gmorhua_Lpl1b            ---MQVKRLIWLCLLDIAILLKSCAALF-NTTNS---------KSTSNT---T-------
XP_005989949.1_Lchalumnae_LPL           ----MTRK--LFILFWIYWVNAVNRTLA-TTIQP---------TVISNS---T-------
XP_028568270.1_Pmuralis_LPL             ----MRRLQFL---------PGTVEEAG-STTDK--------------------------
NP_000228.1_Hsapiens_LPL                ----MESKALLVLTLAVWL-QSLTASRG-GVAAA--------------------------
XP_015135900.1_Ggallus_LPL              ----MERGRGMGKTALLAV-LCLCLRGA-AGSDP---------AEA--------------
XP_002934038.1_Xtropicalis_LPL          ----MSSGEFLTLFLWIHW-LLTCCSGALATTEP---------SKTSPT---T-------
XP_029931044.1_Mmurdjan_Lpl1b           ---MQGKAITWLLIIGIAI-IKSCEALS-NTIEA---------PSLTSP---S-------
XP_013996657.1_Ssalar_Lpl1b             ----MGKEITWLIIIGICI-KSSCESLF-NT-ET---------PALSNG---K-------
XP_036842445.1_Omykiss_Lpl1b            ----MGKEIPWLIIIGICI-KSSCESLF-NTETP----------VLGNG---T-------
XP_005174466.1_Drerio_Lpl1b             ----MGQENFWLLLMGFSL-IASEPIFN-STEEA-----------FASN-----------
XP_042575005.1_Ccarpio_Lpl1b            ----MGTESFWLLLIGFSL--IASEPIS-NSTQ----------VFASNS---S-------
XP_026874726.2_Eelectricus_Lpl1b        ----MGKESVLLLVFGFSL-I-SCDPHI-NSTET---------PFARSI---T-------
XP_034153683.1_Phypophthalmus_Lpl1b     ----MGKENIWLLIIGISV-I-SCKALS-NSTET---------PSASNT---A-------
XP_017351928.1_Ipunctatus_Lpl1b         ----MGKESIWLLIIGISV-I-SCELFS-NSTET----------SSSNT---T-------
XP_049334816.1_Amexicanus_Lpl1b         ----MGKAGIWLLLIGISL-V-SCDSQS-NPTQT---------PADSNT---T-------
XP_036412281.1_Cmacropomum_Lpl1b        ----MGKESIWLLVIGFSL-I-SCDPLS-NSTQA---------PSSSNT---T-------
XP_017552653.2_Pnattereri_Lpl1b         ----MGKESIWLLVIGFSL-I-SCDPLS-NSTQA---------PSSSNT---T-------
XP_042561452.1_Charengus_Lpl1b          ----MGKENLWLLVIGISV-IKSCESLN-TTTES---------VFSSNT---T-------
XP_030630539.1_Cchanos_Lpl1b            ----MGKENIWLLIVGIYF-IKTCAPLS-NTTES---------PLSKNS---T-------
XP_035276326.1_Aanguilla                ----MGKENIGTIIISIYF-IEMCARFS-TTATDE--------TSFSNG---T-------
XP_035276363.1_Aanguilla                --------------MHLIF-KHLSPIFF-ALP--------------GNG---T-------
XP_028672356.1_Ecalabaricus_LPL         ----MGRPSTLLFVVLFYA-LETRADFI-ATTEE---------TPLGNS---T-------
XP_006640052.1_Loculatus_LPL            ----MGKESL-LLFLWIYV-AKTWASLA-ISTEA---------TSLSNT---T-------
XP_023674680.1_Pkingsleyae_Lpl1b        ----MGKQSIYILFFGVCF-IKTCASFS-NTTEATP-------SSTGNT---T-------
XP_036409940.1_Mcyprinoides_Lpl1b       ----MGREHIWLLIVSIYL-VKTCATFS-TPAEETS-------TIPSNS---T-------
NP_001306750.1_Csemilaevis_Lpl1a        ----MGEEKIIFLTVWIIL-GNVLATLS-DTT-----------TEFVNTTVATVSTTPLS
XP_023699829.1_Pkingsleyae_Lpl1a        ----MGKQNIWLLIIWICF-EKTCASLF-NSTES---------PLFSNS---T-------
XP_012683808.2_Charengus_Lpl1a          ----MGKDKLFLLVVWIYL-ENICTVFS-TTTEL---------NYFGNN---T-------
XP_030640550.1_Cchanos_Lpl1a            ----MGKDNSVLLFVWIYF-CNIFVSFA-TSPETTTIEPTPFSATFDNS---T------A
NP_571202.1_Drerio_Lpl1a                MMFNKGRVSSAFLISWMYF-VYISSGLE-TTIDPTAESITLS-DIIGNA---T-------
XP_042568381.1_Ccarpio                  ----MGRVSSACFISWTFF-VYISSGSA-TTIEPTSEF-----PTFNNI---------MD
XP_018919916.2_Ccarpio_Lpl1a            ----MGRVSSACFISWMYL-VYISSGFA-TSIEPASEF-----STFNNI---------ME
XP_042568359.1_Ccarpio                  ----MRHLPCWLLNSLLIW-------FW-TD------------NIMDNG---T-------
XP_026861273.2_Eelectricus_Lpl1a        ----MGSKSVAFLIIWMYL-ARISTGFV-ATAEPATTE-----NTFSNI---T------A
XP_026786482.1_Phypophthalmus_Lpl1a     ----MGRENLVLLIIWMYF-LNLSSSFS-TTFAPPVEPS----AFIGNT---T------T
NP_001316205.1_Ipunctatus_Lpl1a         ----MGRENTVLLIIWMYF-LSLSSSFS-TTIAPTVETS----AFIGNI---T------T
XP_007240188.3_Amexicanus_Lpl1a         ----MGPDSALLLVIWMHC-ASFFCSFA-TTVEPTVEPATET-ATLSNI---T------A
XP_017548830.1_Pnattereri_Lpl1a         ----MGKESAVLLVIWMHF-ASTFSSYA-TTAEPAVET-----DTFSNI---T------A
XP_036442904.1_Cmacropomum_Lpl1a        ----MGKESAFLLVIWMLF-ASTFSSYA-TTAEPVVEA-----DTFSNI---T------A
XP_018583848.2_Sformosus_Lpl1a          ----MGKESIWLLVILVCF-ENTCADFL-NTTAT---------SLPGNT---T-------
XP_035272161.1_Aanguilla                ----MGKQNIWLLVIWIFL-AKKCTSFS-TTTEE---------TLLSNT---T-------
XP_036376712.1_Mcyprinoides_Lpl1a       ----MGKENLWLLVIWIYF-VKTCASFS-TTTEP---------TLFSNT---T-------
XP_030228467.1_Gmorhua_Lpl1a            ----MGKGNFHFLTVWIIL-AQILATFS-SNTT----------VVYDNS---TVTATPVP
XP_010869814.1_Elucius_Lpl1a            ----MGTENIYLITVWIIL-ANICAAFS-TTSEP---------TIFGNN---N-------
XP_014071701.1_Ssalar                   ----MGKENTFLVTVWIIL-ANICVSFS-STPEQ---------TLFGNS---N-------
NP_001118076.1_Omykiss                  ----MGKENTFLVTVWIIL-ANICVSFS-STPEQ---------TLFGNS---N-------
XP_014004153.1_Ssalar                   ----MGKENTYLVTVWIIL-ANICASFS-STPEQ---------TLFGNN---N-------
XP_021435556.2_Omykiss                  ----MGKENTYLVTVWIIL-ANICASFS-STPEQ---------TLFGNN---N-------
XP_003974174.2_Trubripes_Lpl1a          ----MGIKNIFFFTLWIIL-GKFCATFA-SEPDTGTD------AEFVNT---SLPGTPLP
NP_001266682.1_Oniloticus_Lpl1a         ----MGKQNICFLTAWIIL-GKIFATFS-SDLESNT-------TAFANE---TVTIDQLL
NP_001295924.1_Olatipes_Lpl1a           ----MGKELLCFFTLWITL-GNISATFS-SDLESNTT------SLFVNS---TTTAAPLP
XP_029905863.1_Mmurdjan_Lpla            ----MGKENISFLTVWIIL-AKICSAFS-SDPEPT--------SVFANT---T-------
XP_035504156.1_Smaximus_Lpl1a           ----MGKENIRFVTLWIIL-GNTIATFS-TTPGAT--------TVFVNT---TVSTTPLP
XP_040040562.1_Gaculeatus_Lpl1a         ----MGKENICFLSVWIIL-GKIFATFS-SDPELI--------TVFDNT---TVTGTPPP
XP_039666916.1_Pfluviatilis_Lpl1a       ----MGKNNICFLSVWISL-GNIFATFS-SDPELTT-------TVFVNT---TVTASPLP
XP_051233158.1_Dlabrax_Lpl1a            ----MGKENISFLTVWIIL-GKIFATFS-SDPEPTTSNS----TVFVNT---TVTATPLP


XP_007899209.1_Cmilii_LPL               HSPTPGRDFSGIQTRFSLRTPQDPEGDSCFLTPGRVESVAQCGFNISTKTFLIIHGWTVM
XP_030219625.1_Gmorhua_Lpl1b            ---GWFVDYGSIDTKFSLRTAEEPEEDVCYIRPGRQESITECGFNITAQTFAIVHGWTVA
XP_005989949.1_Lchalumnae_LPL           ---GQTKDFSLIETKFSLRTADFPEDDSCYLVPGQQQSIEECNFNLTSKTFVVIHGWTVT
XP_028568270.1_Pmuralis_LPL             ---KW--DFSSIESKFSLRTPGKPDEDTCLIVPGQETSVSQCSFNHTSKTFVVIHGWTVI
NP_000228.1_Hsapiens_LPL                ---DQRRDFIDIESKFALRTPEDTAEDTCHLIPGVAESVATCHFNHSSKTFMVIHGWTVT
XP_015135900.1_Ggallus_LPL              -----EMNFEGIESKFSLRTPAEPDEDVCYLVPGQMDSLAQCNFNHTSKTFVVIHGWTVT
XP_002934038.1_Xtropicalis_LPL          ---LKKTDFNSIESKFSLRTLEEPDDDTCYLVPGQEHTVDQCNFNHTSKTFVVIHGWTVT
XP_029931044.1_Mmurdjan_Lpl1b           ---DAPLDYSAIETKFSVRSPEEPEEDLCYLVPGRPETASECGFNPARKTIAVIHGWAVA
XP_013996657.1_Ssalar_Lpl1b             ---DWILDYTDIETKFSVRSVEEPEEDLCYLVPGKKDTVTQCGFKISLPTFAIIHGWSVA
XP_036842445.1_Omykiss_Lpl1b            ---NWIRDYTDIETKFSVRSVEEPEEDLCYLVPGDKNTVTRCGFNISLPTFAIIHGWSVA
XP_005174466.1_Drerio_Lpl1b             -----FTDYSDIESKFSIRSVEFPDEDLCYLVPGQQDSISDCNFKNDSQTFLIIHGWSVA
XP_042575005.1_Ccarpio_Lpl1b            ---ELTGDYSDIQSKFSIRNPEFPDEDLCYLVPGQRDSISDCNFKNDSQTFLIIHGWSVA
XP_026874726.2_Eelectricus_Lpl1b        ---RWIENHSHIESKFSVRSEEFPDEDMCYLVPGRRESISACNFKTDAQTFLVIHGWSVA
XP_034153683.1_Phypophthalmus_Lpl1b     ---HWRTDFGNIQSKFSLRNAEFPDEDLCYLVPGIRETISDCRFKLDAQTFLVIHGWSVA
XP_017351928.1_Ipunctatus_Lpl1b         ---HWRTDFRSIQSKFSLRIADLPDEDLCYLVPGIRETVSDCRFKLDAQTFLVIHGWSVA
XP_049334816.1_Amexicanus_Lpl1b         ---SWIEDYSDIQSKFSLRHTEFPDEDVCYLVAGDGQTVSDCGFKNDSQTFLVIHGWSVA
XP_036412281.1_Cmacropomum_Lpl1b        ---DWIEDYSNIQSKFSLRHAEFPDEDLCYLVPGETATVLDCNFKPDAQTFLVIHGWSVA
```

```
XP_017552653.2_Pnattereri_Lpl1b      ---DWIEDYSNIQSKFSLRHVEFPEEDVCYLVPGEIGTVLDCNFKPDAQTFLVIHGWSVA
XP_042561452.1_Charengus_Lpl1b       ---LYTEDYSDIETKFSLRTSEYPDEDLCYLILGQQDTISECGFNISSRTFVIIHGWSVA
XP_030630539.1_Cchanos_Lpl1b         ---ETVVDYSDVESKFSVRTAEEPDEDVCYLIPGKPETIAQCNFNTEGQTFLIIHGWSVA
XP_035276326.1_Aanguilla             ---DWLRDYSDIQSKFSLRTCDGPDEDLCYLVPGHPHTAHECQFHAHAQTFMIIHGWTVT
XP_035276363.1_Aanguilla             ---DWLRDYSDIQSKFSLRTCDGPDEDLCYLVPGHPHTAHECQFHAHARTFMIIHGWTVT
XP_028672356.1_Ecalabaricus_LPL      ---EWLADYTSIESKFSLRSPEVPDDDLCYLVPGQINTIEECNFKRTSKTFVVIHGWTVT
XP_006640052.1_Loculatus_LPL         ---DWVVDYSNIQTKFSLRTADEPEDDLCYLVPGQPETISQCNFNPNEQTFLVIHGWTVT
XP_023674680.1_Pkingsleyae_Lpl1b     ---SWIENFSDIESKFSLRTADTPDEDMCYIIPGQPDTIAGCDFKRDSQTFVVIHGWTVT
XP_036409940.1_Mcyprinoides_Lpl1b    ---DWLVDYSDIKTKFSLRTAEEPDDDTCYLVPGQLDTISQCQFNTDVKTFVIIHGWTVT
NP_001306750.1_Csemilaevis_Lpl1a     TTTEWLTDYTDIQSKFSLRSVEEPEDDLCYIVAGKPESIEVCHFNSETQTFIVIHGWTVT
XP_023699829.1_Pkingsleyae_Lpl1a     ---DWIVDFSNIESKFSLRSANVPEDDLCYIVPGEPETISACGFNNKTQTFIVIHGWTVT
XP_012683808.2_Charengus_Lpl1a       ---DWLTDFSDIETKFALRTAEVPDDDLCYIEPGQPESIIQCEFNSEGKTFIVIHGWTVT
XP_030640550.1_Cchanos_Lpl1a         NSTDWMLDFSDIESKFALRTPDMPEDDMCYIVPGQSDTIKECEFNPENKTFIVIHGWTVT
NP_571202.1_Drerio_Lpl1a             ---EWMMDFTDIESKFSFRTLEEPEDDLCYIVPGQPQSIKDCNFNTETKTFIVIHGWTVT
XP_042568381.1_Ccarpio               NGTEWLTDFSDIVSKFSFRTSEEPEDDLCYIVPGQPETIKRCNFNPDNKTFIVIHGWTVT
XP_018919916.2_Ccarpio_Lpl1a         NATEWMTDLSDIESKFSFRTSEEPEDDLCYIVPGQPETIKECNFNPDTKTFIVIHGWTVT
XP_042568359.1_Ccarpio               ---EWMTDFSDIVSKFSFRTSEEPEDDLCYIVPGQPETIKECNFNPDNKTFIVIHGWTVT
XP_026861273.2_Eelectricus_Lpl1a     NSTDRMDDFSDIESRFALRSNNEPDDDLCYIIPGQPQTIVECNFNPDTKTFIVIHGWTVT
XP_026786482.1_Phypophthalmus_Lp     NSTDWMVDFSDIESKFALRTIEEPEDDICYIVPGQRDSIKECNFNPDTKTFIVIHGWTVT
NP_001316205.1_Ipunctatus_Lpl1a      NSTDWMMDFSDIESKFALRTIEEPEEDLCYIAPGQQDTIKECNFNPDTKTFIVIHGWTVT
XP_007240188.3_Amexicanus_Lpl1a      NSTEWMVDFTDIESKFALRTSDEPEDDLCYIVPGQRQTIKECDFNPNEKTFVVIHGWTVT
XP_017548830.1_Pnattereri_Lpl1a      NSTDWMVDFSDIESKFALRTSDEPEDDLCYIVPGQRETIKECNFNPDTKTFIVIHGWTVT
XP_036442904.1_Cmacropomum_Lpl1a     NSTDWMVDFSDIESKFSLRTSDEPEDDLCYIVPGQRETIKECNFNPDSKTFIVIHGWTVT
XP_018583848.2_Sformosus_Lpl1a       ---EGIVDYSNVESKFSLRTAEVPDDDLCYIVPGQPDTVAGCSFNLETQTFVVIHGWTVT
XP_035272161.1_Aanguilla             ---EWITDYSDIESKFSLRTAEDPDDDLCYIVPGQKNTITECEFNLETQTFVVIHGWTVT
XP_036376712.1_Mcyprinoides_Lpl1a    ---DWILDYNDIESKFSLRTAEIPDDDLCYIVPGQPETISQCEFNLQTQTFVIIHGWTVT
XP_030228467.1_Gmorhua_Lpl1a         TSDQWLLDYTGIVSKFSLRTADEPEDDLCYIVPGERDTITECEFNPETQTFVIIHGWTVT
XP_010869814.1_Elucius_Lpl1a         -STKWLEDYTDIVTKFSLRTAEVPDDDLCYIVPGQQLTIPECEFNPETQTFVVIHGWTVT
XP_014071701.1_Ssalar                -STEWLEDYTDIVSKFSLRTAEIPDDDLCYIVPGQPLTIPKCEFNPGYKTFVVIHGWTVT
NP_001118076.1_Omykiss               -STEWLEDYTDIVSKFSLRTAEIPDDDLCYIVPGQPSTIPKCEFNPGYKTFVVIHGWTVT
XP_014004153.1_Ssalar                -STEWLEDYTDILSKFSLRTAAVPDDDLCYIVPGQPLTIPKCEFNSETQTFVVIHGWTVT
XP_021435556.2_Omykiss               -STEWLEDFTDIVSKFSLRTAEVPDDDLCYIVPGQPLTIPECEFNPETQTFMVIHGWTVT
XP_003974174.2_Trubripes_Lpl1a       TTADWINNFTDIVSKFSLRTAEIPDDDMCYIVPGRPETISECHFNPDTQTFVVIHGWTVT
NP_001266682.1_Oniloticus_Lpl1a      NTTEWLTDYTDIVSKFSLRTADTPDDDMCYIVPGRPETIKECNFNSETQTFVVIHGWTVT
NP_001295924.1_Olatipes_Lpl1a        TTTEWMTDYADILSKFSLRTADIPDEDMCYIQAGHPETIKDCQFNAEAQTFIVIHGWTVT
XP_029905863.1_Mmurdjan_Lpla         --DDWITDYTDILSKFSLRTSDVPDDDLCYIVPGRPETIAECEFNPETQTFVVIHGWTVT
XP_035504156.1_Smaximus_Lpl1a        TTAEWITDFTDIVSKFSLRTADIPDDDMCYIFAGRPETIEECKFNASTQSFVVIHGWTVT
XP_040040562.1_Gaculeatus_Lpl1a      TPPGWITDFTDIVSKFSLRTVDIPDDDMCYIVTGSPETVKECEFNPQTQTFIIIHGWTVT
XP_039666916.1_Pfluviatilis_Lpla     TTTEWITDYTDIVSKFSLRTADIPDEDMCYIEAGRPETIKECEFNSETQSFIVIHGWTVT
XP_051233158.1_Dlabrax_Lpl1a         TTAEWITDFTDIVSKFSLRTSEIPDDDMCYIVAGRPETIKECEFNSATQTFIVIHGWTVT
                                            :   : :.*:.*    .  * * :  *   :   * *:    :: ::***:*

XP_007899209.1_Cmilii_LPL            GMFESWIPKLVSALYERERNSNVIVVDWLSRAHQHYTVAADNTRLVGQDIAQFVEWLEEY
XP_030219625.1_Gmorhua_Lpl1b         GIFEGWVSQLVSALLEREPSANVIVVDWLKRAQHHYPNAAQHTQLAGQDLAALINWLELD
XP_005989949.1_Lchalumnae_LPL        GMFESWIPKLVTALYKREPDSNVLVVDWLTRAQQHYPTSAEYTKLVGEDIATFMDWMEEQ
XP_028568270.1_Pmuralis_LPL          GMYESWVPKLVKALYQREPHSNVIVVDWLSRSSLHYPDSAAYTKEVGEDVAKFVDWLEEQ
NP_000228.1_Hsapiens_LPL             GMYESWVPKLVAALYKREPDSNVIVVDWLSRAQEHYPVSAGYTKLVGQDVARFINWMEEE
XP_015135900.1_Ggallus_LPL           GMYESWVPKLVDALYKREPDSNVIVVDWLVRAQQHYPVSAAYTKLVGKDVAMFIDWMEEK
XP_002934038.1_Xtropicalis_LPL       GMFESWVPKLVDALYKREPDSNVIVVDWLTRAQQHYPVSAEYTQLVGQDVASFIDWMDDT
XP_029931044.1_Mmurdjan_Lplb         GFFDSWVYKLVAALFEREPNANIIVVDWLDRAQHHYPNSAENTRLAGQDVAKLINWLEAE
XP_013996657.1_Ssalar_Lpl1b          GLFESWIHKLVAALFERVPNANVIVVDWLDRAQHHYPNSAANTKLVGEDVARLINWLEVD
XP_036842445.1_Omykiss_Lpl1b         GLFASWIHELVAALFERVPNANVIVVDWLDRAQHHYPNSAANTKLVGEDVARLINWLEVD
XP_005174466.1_Drerio_Lpl1b          GLFESWVYKLVTALYDREPSANVIVVDWLDRANKHYPKSAENTRLVGADVAKFVNWLE-E
XP_042575005.1_Ccarpio_Lpl1b         GLFESWVYKLVSALFEREPSANVIVVDWLDRANKHYPKSAENTRLVGADVAKFVNWLE-E
XP_026874726.2_Eelectricus_Lpl1b     GLFESWVSKLVSALLEREPDANVIVVDWLERASHHYPTSAENTRLVGMDVAKFINWLE-S
XP_034153683.1_Phypophthalmus_Lplb   GLFESWIAKLVSALFEREPNANVIVVDWLSRASHHYPTSAENTRLVGRDVAQFIDWLE-V
XP_017351928.1_Ipunctatus_Lplb       GLFESWIAKLVSALFEREPNANVIVVDWLVRASHHYPTSAENTRLVGREVAQFIDWLE-I
XP_049334816.1_Amexicanus_Lpl1b      GLFESWIHKLVSALFEREPGANIIVVDWLDRASHHYPTSAENTRLVGRDVAKFINWLE-T
XP_036412281.1_Cmacropomum_Lpl1b     GLFESWIHKLVSALFEREPTANVIVVDWLDRASHHYPTSAENTRLVGRDVAKFINWLE-G
XP_017552653.2_Pnattereri_Lpl1b      GLFESWIHKLVSALFEREPTANVIVVDWLDRASHHYPTSAENTRLVGRDVAKLINWLE-G
XP_042561452.1_Charengus_Lpl1b       GLFESWVYKFVTAFYEREPSANIIVVDWLDRASQHYPISAENTRLVGKDVAKFINWLE-D
XP_030630539.1_Cchanos_Lpl1b         GLFESWIYKLVSALFEREPTANVIVVDWLHRASQHYPTSAENTKLVGQDVAKFVNWLE-G
XP_035276326.1_Aanguilla             GMFESWVSMLVQALYEREPHANVLVVDWLDRARQHYPTSAANTKLAGRDVAKFINWMEVE
XP_035276363.1_Aanguilla             GMFESWVSMLVQALYEREPHANVLVVDWLDRARQHYPTSAANTKLVGRDVAKFINWMEVE
XP_028672356.1_Ecalabaricus_LPL      GLYESWIAKLVTALFEREPSANVIVVDWLYRAQQHYPTSAAYTKLVGQDLAKFMNWIEVE
XP_006640052.1_Loculatus_LPL         GMFESWVSKLVYALYKREPASNVIVVDWLHRAHQHYPTSAAYTRLVGQDLAHFLNWIEAE
XP_023674680.1_Pkingsleyae_Lpl1b     GMYESWVVKLVKALYEREPAANVLVVDWLARAQQHYPTSAAYTKLVGRDVAKFVSWLQAE
XP_036409940.1_Mcyprinoides_Lpl1b    GLFESWVSKLVVALYEREPAANVIVVDWLNRAQQHYPTSAAYTKLVGQDVAKFINWIETG
NP_001306750.1_Csemilaevis_Lpl1a     GMFESWVPKLVTALYDRVPNGNVIVVDWLARASQHYTTSAEYTKLVGRDVAKFITWIQKE
XP_023699829.1_Pkingsleyae_Lpl1a     GMFESWVPKLVTALYEREPSGNVVVVDWLIRAQQHYPTSAAYTKLVGQDVARFVRWLQAE
XP_012683808.2_Charengus_Lpl1a       GMYESWVPKLVTALYDREPEANVIVVDWLARAQQHYPTSAAYTKLAGRDVAKFVSWLQAL
XP_030640550.1_Cchanos_Lpl1a         GMYESWVPKLVMALHEREPMANVIIVDWLTRAQQHYTTSAAYTKLVGRDVAKFVTWLQGE
NP_571202.1_Drerio_Lpl1a             GMFESWVPKLVTALYEREPSANVIVVDWLSRAQQHYPTSASYTKLVGKDVAKFVNWLQAE
XP_042568381.1_Ccarpio               GMFESWVPKLVTALYEREPTANVIVVDWLSRAQQHYLTSAGYTKLVGRDVAKFVNWLQAE
XP_018919916.2_Ccarpio_Lpl1a         GMFESWVPKLVTALYDREPTANVIVVDWLSRAQQHYPTSAAYTKLVGRDVAKFVNWLQAE
XP_042568359.1_Ccarpio               GMFESWVPKLVTALYEREPTANVIVVDWLSRAQQHYLTSAGYTKLVGRDVAKFVNWLQAE
XP_026861273.2_Eelectricus_Lpl1a     GMYESWVSKLVTALYEREPSANVLVVDWLVRAQQHYPTSAAYTKLVGRDVAKFVNWLQAE
XP_026786482.1_Phypophthalmus_Lpl1a  GMYESWVSKLVTALYEREPVANVIVVDWLVRAQQHYPTSAAYTKLVGRDVAKFVNWLQAE
NP_001316205.1_Ipunctatus_Lpl1a      GMYESWVSKLVTALYEREPTANVIVVDWLDRAQQHYPTSAAYTKPVGRDVAKFVNWLQAE
XP_007240188.3_Amexicanus_Lpl1a      GMYESWVPKLVTALLEREPTANVIVVDWLVRAQQHYPTSAAYTKLVGRDVAKFVNWLQVE
XP_017548830.1_Pnattereri_Lpl1a      GMYESWVPKLVTALYEREPTANVIVVDWLIRAQQHYPTSAAYTKLVGRDVAKFVNWLQAE
XP_036442904.1_Cmacropomum_Lpl1a     GMYESWVPKLVTALYEREPTANVIVVDWLVRAQQHYPTSAAYTKLVGKDVAKFVNWLQAE
XP_018583848.2_Sformosus_Lpl1a       GMFESWVPKLVAALYKREPTANVIVVDWLNRAQQHYPTSAAYTKLVGQDVAKFVNWLENE
XP_035272161.1_Aanguilla             GMYESWVPKLVAALYDREPSANVIVVDWLERASQHYPTSAAYTKLVGHDVGKFVNWLERE
XP_036376712.1_Mcyprinoides_Lpl1a    GMFESWVPKLVSALYEREPSANVIVVDWLTRAQQHYPISAAYTKLVGRDVAKFVKWLETE
XP_030228467.1_Gmorhua_Lpl1a         GMFESWVPKLVSALFNRVPSANVIVVDWLTRANQHYPTSAAYTKLVGADVAKFVTWIQNE
XP_010869814.1_Elucius_Lpl1a         GLFESWVPKLVTALYEREPKANVIVVDWLTRAQQHYSTSAGYTKLVGKDVAKFVNWLQKT
XP_014071701.1_Ssalar                GLFESWVPKLVTALYKREPKANVIVVDWLTRAQQHYLNSAANTKLVGKDVAKFVNWLQKT
```

```
NP_001118076.1_Omykiss                    GLFESWVPKLVTALYKREPKANVIVVDWLTRAQQHYLTSAANTKLVGKDVAKFVNWLQKT
XP_014004153.1_Ssalar                     GLFESWVPKLVTALYEREPKANVIVVDWLTRAQQHYPTSAAYTKLVGRDVAKFVNWLQKT
XP_021435556.2_Omykiss                    GLFESWVPKLVTALYEREPKANVIVVDWLTRAQQHYPTSAAYTKLVGRDVAKFVNWLQKT
XP_003974174.2_Trubripes_Lpl1a            GMFESWVPKLVSALYEREPSANVIVVDWLTRANQHYPMSAAFTKLVGRDVAKFVSWIQNE
NP_001266682.1_Oniloticus_Lpl1a           GMFESWVPKLVSALYDRVPTANVIVVDWLTRANQHYPTSAAYTKLVGRDVAKFVTWIQNE
NP_001295924.1_Olatipes_Lpl1a             GMFESWVPKLVSALYERVPNANVIVVDWLTRASQHYPTSASYTKLVGRDVAKFVTWLQNE
XP_029905863.1_Mmurdjan_Lpl1a             GMFESWVPKLVTALYEREPTANVIVVDWLTRASQHYPSSAADTKLVGRDVAKFVSWIQRE
XP_035504156.1_Smaximus_Lpl1a             GMFESWVPKLVSALYEREPTANVIVVDWLTRANQHYPTSAAYTKLVGRDVAKFVTWIQKE
XP_040040562.1_Gaculeatus_Lpl1a           GMFESWVPKLVSALYEREPSANVIVVDWLTRANQHYPTSAAYTKLVGRDVAKFVTWIQKE
XP_039666916.1_Pfluviatilis_Lpl1a         GMFESWVPKLVSALYDREPGANVIVVDWLTRANQHYPNSAADTKLVGRDVAKFVTWLQKE
XP_051233158.1_Dlabrax_Lpl1a              GMFESWVPKLVSALYEREPSANVIVVDWLTRANQHYPTSAAYTKLVGRDVAKFVTWLQNE
                                          *:: .*:  :* *: .*   .*:::**** *:  **  :*  *. .* ::. :: *::

XP_007899209.1_CmiliiLPL                  SNFPSERIHMLGYSLGAHVAGFAGSHGTNKVGRITGLDPAGPAFEGTEAPYRLSPDDAEF
XP_030219625.1_Gmorhua_Lpl1b              VKYDLSKLHLLGFSLGAHVAGVAGNLAYSKVNRITGMDPAGPHFEYADELRRLSPDDASF
XP_005989949.1_Lchalumnae_LPL             VNYPLERIHLLGYSLGAHVAGIAGSLTNNKINRITGLDPAGPSFEYATDQSRLSPDDADF
XP_028568270.1_Pmuralis_LPL               FSYSLNKVHLLGYSLGAHVAGIAGSLTTNKINRITGLDPAGPNFEYAEASVRLSPDDAVF
NP_000228.1_Hsapiens_LPL                  FNYPLDNVHLLGYSLGAHAAGIAGSLTNKKVNRITGLDPAGPNFEYAEAPSRLSPDDADF
XP_015135900.1_Ggallus_LPL                FNYPLNNVHLLGYSLGAHAAGIAGSLTKKKVNRITGLDPAGPTFEYADAPIRLSPDDADF
XP_002934038.1_Xtropicalis_LPL            IQYPIDNIHILGYSLGAHAAGVAGSLTNKKVNRITGLDPAGPTFEYAENAIILSPDDAEF
XP_029931044.1_Mmurdjan_Lpl1b             LQYDLSKLHLLGYSLGAHVAGVAGNLTNNKVNRITGLDPAGPHFEYANELRRLSPDDATF
XP_013996657.1_Ssalar_Lpl1b               LKYDLKNIHLLGYSLGAHVAGVAGNLTNNKVNRITGMDPAGPRFEYAEHRNRLSPDDATF
XP_036842445.1_Omykiss_Lpl1b              LKYDLKNLHLLGYSLGAHVAGVAGNLTNNKVNRITGMDPAGPRFEYAEHLNRLSPDDATF
XP_005174466.1_Drerio_Lpl1b               LDYPLEKVHLLGYSLGAHVAGVAGNLTNNKVHRITGLDPAGPSFENADILRRLSPDDASF
XP_042575005.1_Ccarpio_Lpl1b              MDYPLERVHLLGYSLGAHVAGVAGNLTNNKVNRITGLDPAGPSFENAESLRRLSPDDASF
XP_026874726.2_Eelectricus_Lpl1b          LGYPLEKLHILGYSLGAHVAGIAGNLTNNKVNRITGLDPAGPTFEHADRLKRLSDDDAEF
XP_034153683.1_Phypophthalmus_Lpl1b       IGYPLEKLHVLGYSLGAHVAGIAGNLTNNKINRITGFDPAGPTFEHADRPNRLSPDDAKF
XP_017351928.1_Ipunctatus_Lpl1b           IGYPLEKLHVLGYSLGAHVAGVAGNLSNNKVNRITGFDPAGPTFEHADRPNRLSPDDAKF
XP_049334816.1_Amexicanus_Lpl1b           LDYPLEKLHVLGYSLGAHVAGVAGHLSNNKVNRITGLDPAGPTFEHADTLKRLSPDDAKF
XP_036412281.1_Cmacropomum_Lpl1b          LGYPLEKLHVLGYSLGAHVAGIAGNLSNKKVNRITGLDPAGPTFEHAHTLKRLSPDDAKF
XP_017552653.2_Pnattereri_Lpl1b           LGYPLEKLHVLGYSLGAHVAGIAGNLSNNKVNRITGLDPAGPTFEHADTLKRLSPDDAKF
XP_042561452.1_Charengus_Lpl1b            LEYPLDKLHLLGYSLGAHVAGVAGNLMNHKVNRITGLDPAGPRFEHAEEISRLSSDDANF
XP_030630539.1_Cchanos_Lpl1b              LEYPLDKIHVLGYSLGAHVAGIAGNLTNSKVNRITGLDPAGPSFENADSLRRLSPDDAKF
XP_035276326.1_Aanguilla                  LQYPLEMVHLLGYSLGAHVAGIAGDLSKNKVYRITGLDPAGPGFEHADAQSSLSPDDAEF
XP_035276363.1_Aanguilla                  LQYPLEMVHLLGYSLGAHVAGIAGDLSKNKVYRITGLDPAGPGFEHADAQSSLSPDDAEF
XP_028672356.1_Ecalabaricus_LPL           GNYTLNNLHLLGYSLGAHVAGIAGSLTNNKVSRITGLDPAGPNFEYAEEKVRLSPDDANF
XP_006640052.1_Loculatus_LPL              LDYPLERSHLLGYSLGAHVAGIAGYLTKKKVNRITGLDPAGPNFEYADEQSSLSPDDAEF
XP_023674680.1_Pkingsleyae_Lpl1b          LKYPWEKIHVLGYSLGAHVAGIAGFLTKQRISRITGLDPAGPGFEYADDQSTLSPDDAEF
XP_036409940.1_Mcyprinoides_Lpl1b         LNYPWEKIHLLGYSLGAHVAGIAGFLSNNKVYRITGLDPAGPGFEHADAQSTLSPDDANF
NP_001306750.1_Csemilaevis_Lpl1a          LQLPWERIHVLGYSLGAHAAGVAGNLTNHKISRITGLDPAGPTFEYADEQSTLSRNDAKF
XP_023699829.1_Pkingsleyae_Lpl1a          LEYPWDRIHLLGYSLGAHVAGIAGSLTSSKIHRITGLDPAGPSFEYADSQSTLSPDDANF
XP_012683808.2_Charengus_Lpl1a            LDYPWDKIHLLGYSLGAHVAGIAGGLTKSQVNRITGMDPAGPTFEYADAQSTLSPDDATF
XP_030640550.1_Cchanos_Lpl1a              VDYPWERIHLLGYSLGAHVAGIAGLLTRSKINRITGLDPAGPSFEYADAQSTLSPDDANF
NP_571202.1_Drerio_Lpl1a                  IDYPWEKLHLLGYSLGAHVAGIAGLLTKHKVNRITGMDPAGPTFEYADSLSTLSPDDANF
XP_042568381.1_Ccarpio                    IDYPWEKLHLLGYSLVLMXQESLGLLTKHKVNRTSGMDPAGPSFEYADAQSTLSPDDALF
XP_018919916.2_Ccarpio_Lpl1a              IDYPWERLHLLGYSLGAHVAGIAGLLTKHKVNRITGMDPAGPSFEYADAQSTLSPDDALF
XP_042568359.1_Ccarpio                    IDYPWEKLHLLGYSLGAHVAGIAGLLTKHKVNRITGMDPAGPSFEYADAQSTLFPDDALF
XP_026861273.2_Eelectricus_Lpl1a          LEYPWEKLHLLGYSLGAHVAGIAGLLTKDKVNRITGLDPAGPSFEYADAQSTLSPDDALF
XP_026786482.1_Phypophthalmus_Lpl1a       LEYPWEKLHLLGYSLGAHVAGIAGLLTRNKVSRITGMDPAGPSFEYADAQSTLSPDDAQF
NP_001316205.1_Ipunctatus_Lpl1a           LEYPWEKLHFLGYSLGAHVAGIAGLLTKNKVSRITGMDPAGPSFEYADAQSTLSPDDAQF
XP_007240188.3_Amexicanus_Lpl1a           IEYPWEKLHLLGYSLGAHVAGIAGLLMKDKVNRITGLDPAGPTFEYADAQSTLSPDDALF
XP_017548830.1_Pnattereri_Lpl1a           LEYPWEKLHILGYSLGAHVAGIAGLLMRNKVNRITGLDPAGPTFEYADAQSTLSPDDALF
XP_036442904.1_Cmacropomum_Lpl1a          LEYPWEKLHILGYSLGAHVAGIAGLLMRNKVNRITGLDPAGPTFEYADAQSTLSPDDALF
XP_018583848.2_Sformosus_Lpl1a            IGYPWEKVHLLGYSLGAHVAGIAGFLTNHKVHRITGLDPAGPGFEFADPQSTLSPDDANF
XP_035272161.1_Aanguilla                  VHYPWEKLHLLGYSLGAHVAGIAGLLTNHKINRITGLDPAGPGFEYADEQSTLSPDDAIF
XP_036376712.1_Mcyprinoides_Lpl1a         LQYPWERIHLLGYSLGAHVAGIAGLLTDHKINRITGLDPAGPSFEYADEQSTLSPDDANF
XP_030228467.1_Gmorhua_Lpl1a              LQLPWDKIHLLGYSLGAHVAGIAGDLTKRQISRITGLDPAGPTFEHAANENILSQEDAKF
XP_010869814.1_Elucius_Lpl1a              LDYPWEKIHLLGYSLGAHVAGIAGFLTDHKVNRITGLDPAGPTFEYADAHSTLSPDDALF
XP_014071701.1_Ssalar                     LDYPWEKIHLLGYSLGAHVAGIAGLLTNHKVSRITGLDPAGPTFEFADAQSTLSPDDALF
NP_001118076.1_Omykiss                    LDYPWEKIHLLGYSLGAHVAGIAGLLTNHKVSRITGLDPAGPTFEFADAQSTLSPDDALF
XP_014004153.1_Ssalar                     LDYPWERIHLLGYSLGAHVAGIAGLLTNHKVSRITGLDPAGPTFEHADTQSTLSPDDALF
XP_021435556.2_Omykiss                    LDYPWEKIHLLGYSLGAHVAGIAGFLTNHKVSRITGLDPAGPTFEHADNQTTLSPDDALF
XP_003974174.2_Trubripes_Lpl1a            LNLPWERIHLLGYSLGAHVAGIAGDLTDHKISRITGLDPAGPTFEHADNQNTLSPDDAKF
NP_001266682.1_Oniloticus_Lpl1a           LQLPWDRVHLLGYSLGAHVAGIAGDLTNHKISRITGLDPAGPTFEHADEQSTLSRGDAQF
NP_001295924.1_Olatipes_Lpl1a             LQLPWDRIHVLGYSLGAHVAGVAGHLTDNKISRITGLDPAGPSFEHADDQSTLSRDDGQF
XP_029905863.1_Mmurdjan_Lpla              LQLPWERIHLLGYSLGAHVAGIAGDLTKQKISRITGLDPAGPTFEHADAESTLSPDDAQF
XP_035504156.1_Smaximus_Lpl1a             LQLPWDKIHLLGYSLGAHVAGVAGDLTNHKISRITGMDPAGPTFEHADDQSTLSRDDAQF
XP_040040562.1_Gaculeatus_Lpl1a           LQLPWERIHLLGYSLGAHVAGVAGDLTDHKISRITGLDPAGPTFEHADNQNTLSKEDAQF
XP_039666916.1_Pfluviatilis_Lpl1a         LQLPWERIHLLGYSLGAHVAGIAGDLTDHKISRITGLDPAGPTFEDAENQNTLSRDDAQF
XP_051233158.1_Dlabrax_Lpl1a              LQLPWERVHLLGYSLGAHVAGVAGDLTDHKISRITGLDPAGPTFEHADNQNTLSRDDAQF
                                                .  *.**:**        *      .: * :*:***** ** :     *   *. *

XP_007899209.1_Cmilii_LPL                 VDALHTFTRGSLGRSIGIQQPVGHVDIYPNGGNFQPGCNLGRVFGNMRSQGV---YAVAE
XP_030219625.1_Gmorhua_Lpl1b              VDVVHTNTKGSPDLSIGIQRPVGHVDIYPNGGMDQPGCTLQHTLQMMATFGL---QKVGL
XP_005989949.1_Lchalumnae_LPL             VDVLHTYTRGSPDRSIGIQKPVGHVDIYPNGGSFQPGCKIGEALRLIAERGF---ADVDQ
XP_028568270.1_Pmuralis_LPL               VDVLHTFTRGSPDRSIGIQKPVGHVDIYPNGGIFQPGCNVAQALLLIAEGGL---QDADQ
NP_000228.1_Hsapiens_LPL                  VDVLHTFTRGSPGRSIGIQKPVGHVDIYPNGGTFQPGCNIGEAIRVIAERGL---GDVDQ
XP_015135900.1_Ggallus_LPL                VDVLHTYTRGSPDRSIGIQKPVGHIDIYPNGGGFQPGCNLGEALRLIAEKGF---SDVDQ
XP_002934038.1_Xtropicalis_LPL            VDVLHTYTRGSPDRSIGIQKPVGHIDIYPNGGSFQPGCNLGEALRLIAEKGF---GDVDQ
XP_029931044.1_Mmurdjan_Lpl1b             VDVLHTNTKGSPDFSIGIQRPVGHVDIYPNGGNEQPGCTFQNAMKMIATFGI---NNVDQ
XP_013996657.1_Ssalar_Lpl1b               VDVLHTNTRGSPDLSIGIQRPVGHVDIYPNGGTAQPGCSFRYTVDMMRSFGL---HNLDQ
XP_036842445.1_Omykiss_Lpl1b              VDVLHTNTRGSPDLSIGIQRPVGHVDIYPNGGTEQPGCSFRHTVDMMRSFGL---HNMDQ
XP_005174466.1_Drerio_Lpl1b               VDVLHTNTRGSPDLSIGIQRPVGHVDIYPNGGTFQPGCSIQHTMKLIATCGI---YNMDQ
XP_042575005.1_Ccarpio_Lpl1b              VDVLHTNTRGSPDLSIGIQRPVGHVDIYPNGGTFQPGCNIQNTMKLIATYGI---YNMDQ
XP_026874726.2_Eelectricus_Lpl1b          VDVLHTNTRGTPDLSIGIQRPVGHVDIYPNGGTFQPGCSLQNAMRMITTYGL---YNVDQ
XP_034153683.1_Phypophthalmus_Lpl1b       VDVLHTNTRGSPDLSIGIQRPVGHVDIYPNGGTFQPGCSLQNTMRMIATYGL---HNMDQ
XP_017351928.1_Ipunctatus_Lpl1b           VDVLHTNTRGSPDLSIGIQRPVGHVDIYPNGGTFQPGCSLQNTMRMIATYGL---YNMDQ
XP_049334816.1_Amexicanus_Lpl1b           VDVLHTNTRGSPDLSIGIQRTVGHVDIYPNGGTFQPGCNIQNAMRMIATHGL---FNMDQ
```

```
XP_036412281.1_Cmacropomum_Lpl1b       VDVLHTNTRGSPDLSIGIQRTVGHVDIYPNGGTFQPGCSLQNTMRMIATYGL---YNMDQ
XP_017552653.2_Pnattereri_Lpl1b        VDVLHTNTRGSPDLSIGIQRTVGHVDIYPNGGTMQPGCSLQNTMKMIATYGL---YNMDQ
XP_042561452.1_Charengus_Lpl1b         VDVLHTNTRGTPDLSIGIQKPVGHVDIYPNGGTYQPGCSLKNTMKMIATYGI---NNMDQ
XP_030630539.1_Cchanos_Lpl1b           VDVLHTNTRGTPDLSIGIQRPVGHVDIYPNGGTFQPGCSLQNTMRMIATYGF---YNMDQ
XP_035276326.1_Aanguilla               VDVLHTNTRGSPGRSIGIQRPVGHVDIYPNGGTSQPGCDLQSAMLKIAMSGI---QNMNQ
XP_035276363.1_Aanguilla               VDVLHTNTRGSPGRSIGIQRPVGHVDIYPNGGTSQPGCDLQSAMLKIAMSGI---QNVNQ
XP_028672356.1_Ecalabaricus_LPL        VDVLHTYTRGSPDRSIGIQKPVGHVDIYPNGGSFQPGCDLHKAMLMIAANGF---AEMDQ
XP_006640052.1_Loculatus_LPL           VDVLHTNTRGSPDLSIGIKRPVGHVDIYPNGGSFQPGCDLHKAMYMIAAAGF---KEMDQ
XP_023674680.1_Pkingsleyae_Lpl1b       VDVLHTNTRGSPDRSIGIQRPVGHVDIYPNGGTFQPGCDLQNTVMMIATSGF---HYMDQ
XP_036409940.1_Mcyprinoides_Lpl1b      VDVLHTNTRGSPGRSIGIQKPVGHVDIYPNGGTFQPGCDLQSTIKMIATSGF---YYMDQ
NP_001306750.1_Csemilaevis_Lpl1a       VDVLHTNTLGSPDRSIGIQRPVGHIDIYPNGGTFQPGCRIQNMLLDIASQGIKGFQNMDQ
XP_023699829.1_Pkingsleyae_Lpl1a       VDVLHTNTRGSPDRSIGIQRPVGHVDIYPNGGGFQPGCDLQNTMRMVATTGF---WNMDQ
XP_012683808.2_Charengus_Lpl1a         VDVLHTNTRGSPDRSIGIQRPVGHVDIYPNGGTFQPGCDLQKTMKMIVSTGF---SNMDQ
XP_030640550.1_Cchanos_Lpl1a           VDVLHTNTRGSPDRSIGIQRPVGHIDIYPNGGTFQPGCDLQNTVKMIAFTGF---QNMDQ
NP_571202.1_Drerio_Lpl1a               VDVLHTNTRGSPDRSIGIQRPVGHIDIYPNGGTFQPGCDLQNTMLMVATTGL---RNMDQ
XP_042568381.1_Ccarpio                 VDVLHTNTRGSPDRSIGIQRPVGHIDVYPNGENIPTGCDLQNTVLMVATSGL---RNMDQ
XP_018919916.2_Ccarpio_Lpl1a           VDVLHTNTRGSPDRSIGIQRPVGHIDIYPNGGTFQPGCDLQNTVLMVATSGL---RNMDQ
XP_042568359.1_Ccarpio                 VDVLHTNTRGSPDRSIGIQRPVGHIDVYPNGGTFQPGCDLQNTVLMVATSGL---RNMDQ
XP_026861273.2_Eelectricus_Lpl1a       VDVLHTNTRGSPDRSIGIQRPVGHIDFYPNGGTFQPGCDLQNTMMMVATTGL---RNMDQ
XP_026786482.1_Phypophthalmus_Lpl1a    VDVLHTNTRGSPDRSIGIQRPLGHVDIYPNGGTFQPGCNLQNTMMMVATTGL---RNMDQ
NP_001316205.1_Ipunctatus_Lpl1a        VDVLHTNTRGSPDRSIGIQRPVGHVDIYPNGGTFQPGCDLQNTMMMVATSGL---RNMDQ
XP_007240188.3_Amexicanus_Lpl1a        VDVLHTNTRGSPDRSIGIQRPVGHVDIYPNGGTFQPGCDLQNTMMMVATTGL---RNMDQ
XP_017548830.1_Pnattereri_Lpl1a        VDVLHTNTRGSPDRSIGIQRPVGHVDIYPNGGTFQPGCDLQNTMMMVATTGL---RNMDQ
XP_036442904.1_Cmacropomum_Lpl1a       VDVLHTNTRGSPDRSIGIQRPVGHVDIYPNGGTFQPGCDLQNTMMMVATTGL---RNMDQ
XP_018583848.2_Sformosus_Lpl1a         VDVLHTNTRGSPDRSIGIQRPVGHVDIYPNGGTFQPGCDLQNTMLMIATTGF---QNMDQ
XP_035272161.1_Aanguilla               VDVLHTNTRGSPDRSIGIQRPVGHVDIYPNGGTFQPGCNLQNTMMLIATSGL---QNMDQ
XP_036376712.1_Mcyprinoides_Lpl1a      VDVLHTNTRGSPDRSIGIQRPVGHVDIYPNGGTFQPGCEFQSTMMMIATTGL---QNMDQ
XP_030228467.1_Gmorhua_Lpl1a           VDVLHTNTRGSPDRSIGIQRSVGHIDIFPNGGTFQPGCDIQNTLMGIASAGIKGLQNMDQ
XP_010869814.1_Elucius_Lpl1a           VDVLHTNTRGSPDRSIGIQRPVGHVDIYPNGGTFQPGCDLQNTMKMIALTGI---HNMDQ
XP_014071701.1_Ssalar                  VDVLHTNTRGSPDRSIGIQRPVGHVDIYPNGGTFQPGCDLQNTMMMIATTGI---RNMDQ
NP_001118076.1_Omykiss                 VDVLHTNTRGSPDRSIGIQRPVGHVDIYPNGGTFQPGCDLQNTMMMIATTGI---RNMDQ
XP_014004153.1_Ssalar                  VDVLHTNTRGSPDRSIGIQRPVGHVDIYPNGGTFQPGCDLQNTMMMIATTGI---HNMDQ
XP_021435556.2_Omykiss                 VDVLHTNTRGSPDRSIGIQRPVGHVDIYPNGGTFQPGCDLQNTMMMIATTGI---HNMDQ
XP_003974174.2_Trubripes_Lpl1a         VDVLHTNTRGSPNRSIGIQRPVGHVDIYPNGGTFQPGCDIQNTLLGIAAEGIKGLHNMDQ
NP_001266682.1_Oniloticus_Lpl1a        VDVLHTNTRGSPDRSIGIQRPVGHIDIYPNGGTFQPGCDIQNTLMGIALEGIKGLQNMDQ
NP_001295924.1_Olatipes_Lpl1a          VDVLHTNTRGSPDRSIGIQRPVGHIDIYPNGGTFQPGCDIQNTLLGIASAGIKGLQNMDQ
XP_029905863.1_Mmurdjan_Lpl1a          VDVLHTNTRGSPDRSIGIQRTVGHIDIYPNGGTFQPGCDIQNTLMGIAQAGIKGFQDMAQ
XP_035504156.1_Smaximus_Lpl1a          VDVLHTNTRGSPDRSIGIQRPVGHIDIYPNGGTFQPGCDIQNTLLGIALEGIKGLQNMDQ
XP_040040562.1_Gaculeatus_Lpl1a        VDVLHTNTRGSPDRSIGIQRAVGHIDIYPNGGTFQPGCDIQNTLMGIALEGIKGLQNMDQ
XP_039666916.1_Pfluviatilis_Lpl1a      VDVLHTNTRGSPGRSIGIQRPVGHIDIYPNGGTFQPGCDISNTLMGIALEGLKGLQNMDQ
XP_051233158.1_Dlabrax_Lpl1a           VDVLHTNTRGSPDRSIGIQRPVGHIDIYPNGGTFQPGCDIQNTLLGIALEGIKGLQNMDQ
                                       **.:** * *: . ****:..:**:*.:***    .** .   .  :   *.

XP_007899209.1_Cmilii_LPL              AIKCQHERA-VHLFIDSLVN-EKYPSMAYRCSNKE-TFEKGMCLSCRKNRCNTMGYDVNR
XP_030219625.1_Gmorhua_Lpl1b           IMGCAHERS-VHLFIDSLVN-RERQSVAYRCSSKD-AFDRGQCLSCRKNRCNTLGYGAHQ
XP_005989949.1_Lchalumnae_LPL          LVKCSHERS-IHLFIDSLLY-EEKPSMAYRCNTKE-AFEKGLCLSCRKNRCNNMGYKVHR
XP_028568270.1_Pmuralis_LPL            LLKCSHERS-IHLFIDSLLY-EEKPIMAYRCNSKE-AFDKGLCLSCRKNRCNNLGYKANR
NP_000228.1_Hsapiens_LPL               LVKCSHERS-IHLFIDSLLN-EENPSKAYRCSSKE-AFEKGLCLSCRKNRCNNLGYEINK
XP_015135900.1_Ggallus_LPL             LVKCSHERS-IHLFIDSLLY-EEKPSMAYRCNTKE-AFEKGLCLSCRKNRCNNLGYKVNR
XP_002934038.1_Xtropicalis_LPL         LVKCSHERS-IHLFIDSLLY-EEKPSMAYRCNSKE-AFEKGLCLSCRKNRCNTLGYKVNK
XP_029931044.1_Mmurdjan_Lpl1b          IVKCAHERS-VHLFIDSLVN-RQQQSMAYRCSSKD-AFNRGLCLSCRKNRCNNLGYGVNK
XP_013996657.1_Ssalar_Lpl1b            ILKCAHERS-VHLFIDSLVN-KQQQSMAYRCNSKD-AFDRGLCLSCRKNRCNNMGFGVNK
XP_036842445.1_Omykiss_Lpl1b           IVKCAHERS-VHLFIDSLVN-KQQQSMAYRCNSKD-AFDRGLCLSCRKNRCNNMGFGVNK
XP_005174466.1_Drerio_Lpl1b            IVKCSHERS-IHLFIDSLVN-QAYQSWAFRCASRD-SFNKGLCLSCRKNRCNTLGYNVKK
XP_042575005.1_Ccarpio_Lpl1b           IVKCSHERS-IHLFIDSLVN-RAYQSWAYRCSSRD-AFNKGLCLSCRKNRCNKLGYGVNK
XP_026874726.2_Eelectricus_Lpl1b       LVKCSHERS-VHLFIDSLVN-WDRQSLAYRCSSKE-AFSKGLCLSCRKNRCTRLGYNVSK
XP_034153683.1_Phypophthalmus_Lpl1b    LVKCSHERS-VHLFIDSLVN-QDQQSLAYRCSSKE-AFYKGLCLSCRKNRCNKLGYNVNK
XP_017351928.1_Ipunctatus_Lpl1b        LVKCSHERS-VHLFIDSLVN-KDQQSLAYRCSSKE-AFYKGLCLSCRKNRCNKLGYNINK
XP_049334816.1_Amexicanus_Lpl1b        LVKCSHERS-VHLFIDSLVN-QEQQSMAYRCNSKE-AFYKGMCLSCRKNRCNKVGYNVNK
XP_036412281.1_Cmacropomum_Lpl1b       LVKCSHERS-VHLFIDSLVN-QDQQSLAYRCSSKE-AFYKGLCLSCRKNRCNKLGYNVNK
XP_017552653.2_Pnattereri_Lpl1b        LVKCSHERS-VHLFIDSLVN-QDQQSLAYRCSSKE-AFYKGLCLSCRKNRCNKLGYNVNK
XP_042561452.1_Charengus_Lpl1b         LVKCSHERS-IHLFIDSLVN-LQHQSLAYRCSSKE-AFYKGLCLSCRKNRCNKLGYGVNK
XP_030630539.1_Cchanos_Lpl1b           LVKCSHERA-VHLFIDSLVN-QAQQSWAYRCGSKD-SFNKGLCLSCRKNRCNKLGYSVNK
XP_035276326.1_Aanguilla               LMKCSHERS-VLLFVDSLLN-SEQQSTAFLCSSRE-TFSRGLCLSCRGNRCAKLGYGARP
XP_035276363.1_Aanguilla               LMKCSHERS-VLLFFDSLLN-SEQQSTAFLCSSRE-TFSRGLCLSCRGNRCAKLGYGARP
XP_028672356.1_Ecalabaricus_LPL        IVKCSHERS-IHLFIDSLLN-EEKQSMAYRCNAKE-TFEKGLCLSCRKNRCNTLGYEINK
XP_006640052.1_Loculatus_LPL           LVKCSHERS-VHLFIDSLLN-EHKQSLAFRCGAKE-TFDRGLCLSCRKNRCNKVGYEVKK
XP_023674680.1_Pkingsleyae_Lpl1b       IVKCSHERS-IHLFIDSLVN-QEKQSLAFRCGTKE-AFNNGLCLSCRKNRCNKLGYEVSK
XP_036409940.1_Mcyprinoides_Lpl1b      IVKCSHERS-IHLFIDSLVN-EEQQSLAYRCSSKE-TFNQGLCLSCRKNRCTKMGYDVQK
NP_001306750.1_Csemilaevis_Lpl1a       LVKCSHERS-IHLFIDSLLN-TQQQSMAYRCNSNQ-AFNKGMCLTCRKNRCNKIGYNINK
XP_023699829.1_Pkingsleyae_Lpl1a       IVKCSHERS-IHLFIDSLVN-EQKQSTAYRCPSKE-AFDKGLCLSCRKNRCNKLGYEVNK
XP_012683808.2_Charengus_Lpl1a         IVKCSHERS-IHLFIDSLLNAHAHQTMAYSCSNKE-SFEKGVCLSCRKNRCNKLGYEINR
XP_030640550.1_Cchanos_Lpl1a           IVKCSHERS-IHLFIDSLVN-EHMQSFAYRCNSKD-AFNKGMCLSCRKNRCNKVGYGVNK
NP_571202.1_Drerio_Lpl1a               IVKCSHERS-IHLFIDSLVN-QDHESMAFRCSSRD-SFNKGMCLSCRKNRCNKVGYAVNK
XP_042568381.1_Ccarpio                 IVKMLHECAPIHLFIDSLLN-QEQESLAYRCSSKKTASNKGMCLSCRKNRCNKVGYGVNK
XP_018919916.2_Ccarpio_Lpl1a           IVKCSHERS-IHLFIDSLVN-QDQESMAYRCSSKD-SFNKGMCLSCRKNRCNKVGYGVNK
XP_042568359.1_Ccarpio                 IVKCSHERS-IHLFIDSLLN-QEQESLAYRCSSKD-SFNKGMCLSCRKNRCNKVGYGVNK
XP_026861273.2_Eelectricus_Lpl1a       IVKCSHERS-IHLFIDSLIN-SEQESMGYRCSSKE-TFNKGVCLSCRKNRCNKVGYGVNK
XP_026786482.1_Phypophthalmus_Lpl1a    IVKCSHERS-IHLFIDSLVN-MKQQSMAYRCSSKE-TFNKGVCLSCRKNRCNKAGYEVNK
NP_001316205.1_Ipunctatus_Lpl1a        IVKCSHERS-IHLFIDSLVN-MKQQSMAYRCSSKE-TFNKGVCLSCRKNRCNKVGYEVNK
XP_007240188.3_Amexicanus_Lpl1a        IVKCSHERS-IHLFIDSLVN-AEQPSMAFRCSSKE-IFNKGVCLSCRKNRCSKAGYDVTK
XP_017548830.1_Pnattereri_Lpl1a        IVKCSHERS-IHLFIDSLIN-MEQQSMAYRCSSKE-TFNKGVCLSCRKNRCNKVGYDVTK
XP_036442904.1_Cmacropomum_Lpl1a       IVKCSHERS-IHLFIDSLIN-MEQQSMAYRCSSKE-TFNKGVCLSCRKNRCNKVGYDVTK
XP_018583848.2_Sformosus_Lpl1a         IVKCSHERS-IHLFIDSLVN-KEKQSMAYRCNKKE-AFDKGLCLSCRKNRCNTLGYQVNK
XP_035272161.1_Aanguilla               IVKCSHERS-IHLFIDSLVN-KQQESMAYRCSSKE-AFNKGLCLSCRKNRCNKVGYGIHK
XP_036376712.1_Mcyprinoides_Lpl1a      IVKCSHERS-IHLFIDSLVN-EQHQSLAYRCSSKE-AFNKGLCLSCRKNRCNKLGYEVNK
XP_030228467.1_Gmorhua_Lpl1a           LVKCSHERS-IHLFIDSLVN-ADQQSMAYSCNSQS-AFNKGLCLSCRKNRCNNLGYNVKK
XP_010869814.1_Elucius_Lpl1a           IVKCSHERS-IHLFIDSLVNGQDHQSMAYRCSSKE-AFMKGMCLNCRKNRCNKVGYNVVA
```

```
XP_014071701.1_Ssalar                LVKCSHERS-IHLFIDSLVNAAEHQTMAYRCSSKE-AFMKGMCLNCRKNRCNKVGYGVNK
NP_001118076.1_Omykiss               LVKCSHERS-IHLFIDSLVNAAEHQTMAYRCSSKE-AFMKGMCLNCRKNRCNKVGYGVNK
XP_014004153.1_Ssalar                IVKCSHERS-IHLFIDSLVNAAEHQSMAYRCSSKE-AFMKGMCLNCRKNRCNKVGYGVNK
XP_021435556.2_Omykiss               IVKCSHERS-IHLFIDSLVNAAEHQTMAYRCSSKE-AFMKGMCLNCRKNRCNKVGYGVNK
XP_003974174.2_Trubripes_Lpl1a       LVKCSHERS-IHLFIDSLIN-TEQQSVAYRCNSKE-AFNKGLCLSCRKNRCNKLGYNVNQ
NP_001266682.1_Oniloticus_Lpl1a      LVKCSHERS-IHLFIDSLLN-IEQQSVALRCNSKD-TFNKGMCLSCRKNRCNKIGYNVKK
NP_001295924.1_Olatipes_Lpl1a        LVKCSHERS-IHLFIDSLVN-TQHQSMAYRCNSKE-AFNKGVCLSCRKNRCNKLGYNINK
XP_029905863.1_Mmurdjan_Lpl1a        IVKCSHERS-IHLFIDSLVN-TGQQSMAYRCNSKE-AFNKGLCLSCRKNRCNKLGYNVNK
XP_035504156.1_Smaximus_Lpl1a        LIKCSHERS-IHLFIDSLLN-TQQQSMAYRCNSKE-AFNKGLCLNCRKNRCNKLGYNINK
XP_040040562.1_Gaculeatus_Lpl1a      LVKCSHERS-IHLYIDSLLN-TQRQTMAYRCNSRE-TFNKGMCLSCRKNRCNKLGYNINK
XP_039666916.1_Pfluviatilis_Lpl1a    LVKCSHERS-IHLFIDSLLN-TQQQSLAYRCNSRE-AFNKGLCLSCRKNRCNKLGYNINK
XP_051233158.1_Dlabrax_Lpl1a         LVKCSHERS-IHLFIDSLLN-TQQQSMAYRCNSKD-AFNKGLCLSCRKNRCNTLGYNINK
                                      :   ** : : *:.***:        .  *  ..    .* **.** ***   *:

XP_007899209.1_Cmilii_LPL            VRSKRSGRMYLKTRADMPFRVYHYQLKIHFFRTINRTEKGTTFLISLFGTRNETGALAVE
XP_030219625.1_Gmorhua_Lpl1b         QRTTRAAKMYLKTTDRTPFKVFHYQIKVHLRSWKSLTFSQQPLLVSLHGTLGDKENIFVI
XP_005989949.1_Lchalumnae_LPL        IRAKRSSKMYLKTRSQAPYKVFHYQIKLHFFAPENWTKTDQSFLVSLYGTNNEAEEIAVL
XP_028568270.1_Pmuralis_LPL          VRSKRGTKMYLKTRSQMPYKVYHYQVKIHFFAKVNTTKKDQQFRLSFYGTKDESENIAIS
NP_000228.1_Hsapiens_LPL             VRAKRSSKMYLKTRSQMPYKVFHYQVKIHFSGTESETHTNQAFEISLYGTVAESENIPFT
XP_015135900.1_Ggallus_LPL           VRTKRNTKMYLKTRAQMPYKVFHYQVKIHFFGKTNVTKVDQPFLISLYGTLDESENIPFT
XP_002934038.1_Xtropicalis_LPL       VRGKRSTKMYLKTRAQMPFKVFHYQVKVHFFAKKNLTVTDQPFLVSLYGTQGESVNMPIT
XP_029931044.1_Mmurdjan_Lpl1b        VRRARSTKMYLKTGAQMPFKVFHYQVKAHFFSQKNLILSQQPILVSLYGTHGEKENIPLI
XP_013996657.1_Ssalar_Lpl1b          IRTTRSAKMYLKTGASMPFKVFHYQIKFHVFNQQNLSLNEQPFIVSLYGTHGEKEAVQLI
XP_036842445.1_Omykiss_Lpl1b         IRTTRSAKMYLKTGASMPFKVFHYQMKFHVFKQQNLSLNEQPFIVSLYGTHGEKEDVQLL
XP_005174466.1_Drerio_Lpl1b          IRSTRSTKMYLKTREMMPFKVFHYQIKMHMFSDKNMTLLEQPMKVSLFGTIDERNDISVI
XP_042575005.1_Ccarpio_Lpl1b         IRRARSAKMYLKTRDMMPFRVFHYQIKLHLFSDKNMTLLEQPMKVSLFGTTDERDDIAVT
XP_026874726.2_Eelectricus_Lpl1b     VRSTRSARMYLKTRDVMPYKVFHYQIKVHLFSQKDMTLESQPLKISLFGIRDERDNIPVV
XP_034153683.1_Phypophthalmus_Lpl1b  VRTARSTRMYLKTREMMPYKVFHYQIKAHLFSLNNLALDNQPVKISLYGTHDEKDNIPIV
XP_017351928.1_Ipunctatus_Lpl1b      VRTARSTRMYLKTREMMPYKVFHYQIKVHLFSHSNLALDNQPVKISLYGTHDEKDGIPIV
XP_049334816.1_Amexicanus_Lpl1b      IRSTRSTKMYLKTREMMPFKVYHYQIKLHLFSLKNMTLENQPVKISLYGTHDERDDIPVV
XP_036412281.1_Cmacropomum_Lpl1b     VRTTRSAKMYLKTREMMPYKVFHFQIKLHLFSQKNLTMDNQPVKISLFGTRDQRDDIPVV
XP_017552653.2_Pnattereri_Lpl1b      VRTTRSAKMYLKTREMMPYKVFHFQIKLHLFSQKNMALENQPVKISLFGTRDQRDDIPVV
XP_042561452.1_Charengus_Lpl1b       IRTTRSAKMYLKTRELMPYKVFHYQVKVHVFGQKNFSVVEQPMLVSLYGTHGDQEGIPVT
XP_030630539.1_Cchanos_Lpl1b         IRTTRSTKMYLKTRAMMPYKVFHYQIKVHIFNQKNVSLVEQPLKVSLYGTHGEREEITVT
XP_035276326.1_Aanguilla             VRARRSLTMYLHTRGVMPYKVFHFQVKVHFFSQDNVTFRDQPLKVSLFGTLGEKEDISLI
XP_035276363.1_Aanguilla             VRARRSLTMYLHTRGVMPYKVFHFQVKVHFFSH---------------------------
XP_028672356.1_Ecalabaricus_LPL      VRSKRSSRMYLKTREVMPYKVFHYQLKVHFFSQENITFTDQPVQVSLYGTHDEAQNIALV
XP_006640052.1_Loculatus_LPL         VRSRRSSRMYLKTREIMPYKVFHYQIKLHFFSQKNISFTVQPVQVSLHGTQNETENIALV
XP_023674680.1_Pkingsleyae_Lpl1b     VRSRRSVRMFLKTREAMPFKVFHYQVKLHLFSQQNQTFIEQPIKITLLGTNGVKEDIALV
XP_036409940.1_Mcyprinoides_Lpl1b    VRSRRSATMYLKTREAMPYKVFHYQVKVHFFNDRNATFKDEPLKVSLIGVLDKKEDISLV
NP_001306750.1_Csemilaevis_Lpl1a     VRTARSTTMYLKTRGEMPFKVFHYQVKMHVFSKDKLNFTEQPIIISLYGTEGEKEGISFV
XP_023699829.1_Pkingsleyae_Lpl1a     VRSRRNAKMYLKTRQMMPFKVFHYQVKMHLFSKDKLQYSEQPWKVSLHGVHGETEDISIL
XP_012683808.2_Charengus_Lpl1a       VRTRRSSKMYLKTREMMPFKVFHYQVKLHFFSKDALSFTEQPIRVSLYGTLGEREDLTHV
XP_030640550.1_Cchanos_Lpl1a         VRARRSSKMYLKTRGIMPYKVFHYQVKVHFFGKDKMSFTEQPMQISLYGLHGEKENIPYV
NP_571202.1_Drerio_Lpl1a             IRTRRSSKMYMKTREMMPYKVFHYQVKVHFFGKTQLSYTDQPMKISLYGIHGEKENIPYI
XP_042568381.1_Ccarpio               IRTRRSSKMYMKTRDVMPYKVFQYQVKVHFFGKTKLSYTDQPMKISLYGIHGEKENIPYI
XP_018919916.2_Ccarpio_Lpl1a         IRTRRSSKMYMKTRNVMPYKVFHYQVKVHFFGKTKLSYTDQPIKISLYGIHGEKENIPYI
XP_042568359.1_Ccarpio               IRTRRSSKMYMKTRDVMPYKVFHYQVKVHFFGKTKLSYTDQPMKISLYGIHGEKENIPYI
XP_026861273.2_Eelectricus_Lpl1a     VRSRRSSRMYMKTRDMMPFKVFHYQVKVHFFGKTKISYNDQPMKISLYGYSGEKENIPYV
XP_026786482.1_Phypophthalmus_Lpl1a  VRSRRSSKMYMMTREMMPYKVFHYQVKVHFFSKSKISYTDQPVKISLYGYSGEKENIPYI
NP_001316205.1_Ipunctatus_Lpl1a      VRSRRSSKMYTKTREMMPYKVFHYQVKVHFFSNSKISYTDQPMKISLYGYSGEKENIPYI
XP_007240188.3_Amexicanus_Lpl1a      VRSRRSSKMYMKTRDMMPYKVFHYQVKVHFFSKNKLSYTDQPMKISLYGYAGEKENIPYV
XP_017548830.1_Pnattereri_Lpl1a      VRSRRNSKMYMKTRDMMPYKVYHYQVKVHFFSQSKISYTDQPMKISLYGYAGEKENIPYV
XP_036442904.1_Cmacropomum_Lpl1a     VRSRRSSKMYMKTRDMMPYKVYHYQVKVHFFSQSKISYTDQPMKISLYGYAGEKENIPYV
XP_018583848.2_Sformosus_Lpl1a       VRSRRSAKMYLKTREMMPFKVFHYQVKVHLFSQDKLEFTEQPMKMSLYGTDGEAEAIAFT
XP_035272161.1_Aanguilla             VRSRRNAKMYLKTREIMPFKVFHYQVKVHFFSQDKLSFTEQPLLVSLFGTEGEKEDIPFT
XP_036376712.1_Mcyprinoides_Lpl1a    IRSRRSSKMYLKTREMMPYKVFHYQLKVHFFSKDKLSFTEQPIKVSLYGTHSEKEDISVT
XP_030228467.1_Gmorhua_Lpl1a         VRSTRSTKMYLKTRDMMPYKVFHYQVKMHFFSEKKLSFTEQPLMISLFGTHGERENIPFQ
XP_010869814.1_Elucius_Lpl1a         VRLPRSTKMFLKTREMMPFKLFHFQVKIHFFSSEKLSYTEQPMKISLYGTHDEKTDIPYI
XP_014071701.1_Ssalar                VRLPRSTKMYLKTREMMPFKLFHYQVKVHFFSSEKLAYTEQPMKISLYGTHDEKTDIPYT
NP_001118076.1_Omykiss               VRLPRNTKMYLKTREMMPFKLFHYQVKVHFFSSEKLAYTEQPMKISLYGTHDEKIDIPYT
XP_014004153.1_Ssalar                VRLPRSTKMYLKTREMMPFKLFHYQVKVHFFSSEKLDYTEQPMKISLYGTHDEKTDIPYI
XP_021435556.2_Omykiss               VRLPRSTKMYLKTRETMPFKLFHYQVKVHFFSSEKLDYTEQPMKISLYGTHDEKTDIPYI
XP_003974174.2_Trubripes_Lpl1a       VRTTRGTKLYLKTREMMPYKVFHYQVKVHFFSETQQSFTEQPMKISLYGTHGEKEDIPFV
NP_001266682.1_Oniloticus_Lpl1a      VRTARSTRMYLKTRGMMPFKVFHYQVKAHFFSQNQQSFTEQPMKISLYGTNGEKEDIAFV
NP_001295924.1_Olatipes_Lpl1a        VRTARSTKMYLKTRGMMPYKVFHYQVKAHFFSKSEQSFTEQPMKISLFGTHGETEDIAFV
XP_029905863.1_Mmurdjan_Lpla         VRTPRSIKMYLKTREMMPYKVFHYQVKVHFFSTEELNYKEQPLKISLYGTHGEKEDIPFV
XP_035504156.1_Smaximus_Lpl1a        VRTTRSTKMFLKTRDMMPYKVFHYQVKMHFFSKDPLSFTEQPLKISLYGTNAEKEDIPFV
XP_040040562.1_Gaculeatus_Lpl1a      VRSTRSAKMYLKTREISPYKVFHYQVKVHLFNKDPLSFTEQPMKISLYGTHGEKEGISFV
XP_039666916.1_Pfluviatilis_Lpl1a    VRMTRSVKMYLKTREMMPYKVFHYQVKVHFFSKDSLSFTEQPMKISLYGTHGEKEDISFV
XP_051233158.1_Dlabrax_Lpl1a         VRMARSTKMYLKTRDMMPYKVFHYQVKVHIFSKDSLSFTEQPMKISLYGTHGEKEDIPFV
                                      *  *   ::  *    *:.::::*:* *.

XP_007899209.1_Cmilii_LPL            VPQISSNQTYSFLVHTDVDIGE-LLIVKLQWETASSVWSTIVGTVTNPWSVWDFWNSREE
XP_030219625.1_Gmorhua_Lpl1b         LEELSANHTASFLVTSDVEVGE-LLRVNLQWESDSVF----------SF-----FN----
XP_005989949.1_Lchalumnae_LPL        MPEVGSNKTYSFLTYTNIDIGD-LLMLKLKWESDSFF----------SWSEW--WK----
XP_028568270.1_Pmuralis_LPL          LPEISTNKTHSFLIYTEVDIGD-LLIVKIQWEKGSFL----------DWADW--WG----
NP_000228.1_Hsapiens_LPL             LPEVSTNKTYSFLIYTEVDIGE-LLMLKLKWKSDSYF----------SWSDW--WS----
XP_015135900.1_Ggallus_LPL           LPEVSSNKTFSFLIYTEVDIGD-LLMLKLQWEKDTFF----------SWSDW--WT----
XP_002934038.1_Xtropicalis_LPL       IPEISTNKTYSYLIYTELDIGD-LLMLKIKWEKDSYF----------SWSDW--FT----
XP_029931044.1_Mmurdjan_Lpl1b        LPELNTNTTVSFLLTTDVDIGE-LLMVKMLWESDSFF----------SY-----FR----
XP_013996657.1_Ssalar_Lpl1b          LSNVTTNTTVSFLLTTDVDLGE-LLRVTLQWDSDSYF----------SF-----FN----
XP_036842445.1_Omykiss_Lpl1b         LSNVTTNTTVSFLLTTDVDLGE-LLRVTLQWDSDSYF----------SF-----FN----
XP_005174466.1_Drerio_Lpl1b          VPSMSTNSTVSFLVTSDRDIGD-LLMLKLHWEKDSYL------------YSF--FS----
XP_042575005.1_Ccarpio_Lpl1b         VPSMSTNSTVSFLLTSDKDIGE-LLMLSLHWEKDSYL------------YSF--FS----
XP_026874726.2_Eelectricus_Lpl1b     VSRMITNTTVSFLVTCDTDIGE-LLMLSVQWEKDSFL------------SGF--FS----
XP_034153683.1_Phypophthalmus_Lpl1b  VPSMITNTTISFLVTSDVDVGD-LLMLKIQWESDSYL------------PRF--FS----
XP_017351928.1_Ipunctatus_Lpl1b      VPSMITNTTISFLVTSDVDVGD-LLMLKIQWESDSYL------------PSF--FS----
```

```
XP_049334816.1_Amexicanus_Lpl1b       VPSMITNTTISFLVTSDVDVGE-LLMLKVQWETDSYL------------PNF--FS----
XP_036412281.1_Cmacropomum_Lpl1b      VPSMITNMTISFLVTSDVDVGE-LLMLKVQWETDSYL------------PSF--FS----
XP_017552653.2_Pnattereri_Lpl1b       VPSMITNTTISFLVTSDVDVGE-LLILKVQWETDSYL------------PSF--FS----
XP_042561452.1_Charengus_Lpl1b        VPNMSTNTTISFLVVSDVDVGE-LLMVKLKWEKDSYL------------SGF--FG----
XP_030630539.1_Cchanos_Lpl1b          MPGMITNTTISFLVTSDVDVGE-ILMIKMQWEKDSYF------------SGF--FG----
XP_035276326.1_Aanguilla              LPTMQGNSTASFLVTSDVEVGE-LLRVELQWVKDYVF----------KF--WEHPG----
XP_035276363.1_Aanguilla              -----------------------------FFER---------------------YQ----
XP_028672356.1_Ecalabaricus_LPL       LPKLSANKTFSFLVTTDINIGD-LLMVKLMWEKDSYF----------SWTDW--WK----
XP_006640052.1_Loculatus_LPL          LPAMSTNKTISFLLTTDVNIGE-LLMVKMKWEKDSYF----------SWSDW--WG----
XP_023674680.1_Pkingsleyae_Lpl1b      VPQLSTNSTISFLLTSDVDIGD-LLMVKMVWEKDSFL----------SLSSW--WS----
XP_036409940.1_Mcyprinoides_Lpl1b     LPVMHTNTTVSFLLTSDVDIGE-LREVKLQWEKDSVF----------SFSNW--WS----
NP_001306750.1_Csemilaevis_Lpl1a      LPDINYNSTLSFLITTDTNIGD-LRMVKLRWEKDSLF----------SWSDL--WR----
XP_023699829.1_Pkingsleyae_Lpl1a      VPALNSNATISFLLITEVDIGD-LIMVNIKWEKDSLF----------TLYDF--WG----
XP_012683808.2_Charengus_Lpl1a        LPELKTNTTVSYLLTTDMDIGE-LLMVKMRWDKDSYF----------SFSDW--WS----
XP_030640550.1_Cchanos_Lpl1a          LPSLATNTTVSFLLTTDVDIGE-LLMVKIQWEKDSLF----------RWS-W--WG----
NP_571202.1_Drerio_Lpl1a              MPTLNTNSTVSFLLTTDADIGE-LLMVKLLWEKDTLI----------SWP-W--WN----
XP_042568381.1_Ccarpio                MPALNTNTTVSFLLTTDVDIGEQLLIXKLFWEKDTLI----------SWP-W--WN----
XP_018919916.2_Ccarpio_Lpl1a          LPALNTNTTVSFLLTTDTDIGE-LLMVKLLWEKDTLI----------SWP-W--WN----
XP_042568359.1_Ccarpio                MPALNTNTTVSFLLTTDVDIGE-LLIVKLFWEKDTLI----------SWP-W--WN----
XP_026861273.2_Eelectricus_Lpl1a      MPSLNTNTTVSFLLTTDVDIGE-LLMVKLVWEKDMLI----------SWP-W--WS----
XP_026786482.1_Phypophthalmus_Lpl1a   IPALKTNITISFLLTTDVDIGE-LLMVKLLWEKDTIL----------SWP-W--WN----
NP_001316205.1_Ipunctatus_Lpl1a       MPALKTNTTISFLLTTDVDIGE-LLMVKLLWEKDTIL----------SWP-W--WN----
XP_007240188.3_Amexicanus_Lpl1a       MPTLSSNSTVSFLLTTDVDIGE-LVMVKIVWEKDTLI----------SWP-W--WN----
XP_017548830.1_Pnattereri_Lpl1a       MPAMKTNTTVSFLLTTDVDIGE-LMMVKLVWEKDTII----------GWP-W--WN----
XP_036442904.1_Cmacropomum_Lpl1a      MPTMNTNTTVSFLLTTDVDIGE-LMIVKLVWEKDTII----------SWP-W--WN----
XP_018583848.2_Sformosus_Lpl1a        LPLLYSNTTVSFLLTTDVNVGD-LLKVTLQWENDSLF----------SLSSW--WS----
XP_035272161.1_Aanguilla              MPYLYTNTTISFLLTTDVDIGE-LLMVKMKWEKDSYF----------SLSDW--WG----
XP_036376712.1_Mcyprinoides_Lpl1a     LPQLTANTTISFLLTTDVDIGE-LLMVKMRWEKDSYF----------SLSDW--WG----
XP_030228467.1_Gmorhua_Lpl1a          IPTLEGNSTVSFLLTTDTEIGD-LLIVKLRWEKDNFF----------SWSDW--WG----
XP_010869814.1_Elucius_Lpl1a          MPQLNSNSTVSFLLTTEVDIGE-LLMVKLRWEKDAYF----------SWSDW--WG----
XP_014071701.1_Ssalar                 MPFLNTNSTVSFLLTTDVDVGE-LLMVKLRWEKDAYF----------SWSDM--WG----
NP_001118076.1_Omykiss                MPFLNTNSTVSFLLTTDVDVGE-LLMVKLRWEKDAYF----------SWSDI--WG----
XP_014004153.1_Ssalar                 MPFLNTNSTVSFLLTTDVDIGE-LLMVKLRWEKDAYF----------SWSDW--WG----
XP_021435556.2_Omykiss                MPFLNTNSTVSFLLTTDVDIGE-LLMVKLRWEKDAYF----------SWSDW--WG----
XP_003974174.2_Trubripes_Lpl1a        LPELNSNTTVSFLLTTDVDLGD-LMIVKLRWEKDAII----------SWSDW--WG----
NP_001266682.1_Oniloticus_Lpl1a       LPSLNSNTTQSFLITTDTDIGD-LMIVKLRWERDAFI----------SWSNW--WG----
NP_001295924.1_Olatipes_Lpl1a         LPELSCNNTVSFLITTDVDIGE-LMIVKLRWEKDAIL----------SWSDW--WG----
XP_029905863.1_Mmurdjan_Lpl1a         LPELNGNSTVSFLLTTDVDIGD-LMIVKLRWEKDAFF----------SWSDW--WG----
XP_035504156.1_Smaximus_Lpl1a         MPVLNGNTTLSFLITTDVAIGD-LMIVKLRWEKDSII----------SWSDF--WG----
XP_040040562.1_Gaculeatus_Lpl1a       LPVLSGNTTLSFLITTDVDVGD-LMIVKLRWEKDTII----------SWTDW--WG----
XP_039666916.1_Pfluviatilis_Lpl1a     LPVLSGNTTFSFLITADEDIGD-LMIVKLRWEKDTLI----------SWSDW--WG----
XP_051233158.1_Dlabrax_Lpl1a          LPDLTGNTTLSFLITTDVDIGE-LMFVKLRWEKDTII----------SWSNW--WG----
                                                                    :

XP_007899209.1_Cmilii_LPL             SSKDLEIRKIRVKSGETQKKIVFCPK-GAAFVKI-QPTEEVIFVKCPSGWMSNSRR----
XP_030219625.1_Gmorhua_Lpl1b          -TNQFTVHRMRVKAGETQAKIKFTAR-GADLAQLVQGGEAAVFVRTKENQTNRRHKR---
XP_005989949.1_Lchalumnae_LPL         -SYAFDIRKIRVKSGETQHKLVFCSK-DSAFAHLEKGKESVVFVKCPDGHVSKRHSRHYR
XP_028568270.1_Pmuralis_LPL           -TPEFGIQKVRVKAGETQKKLVFCSR-DG-FSYLKKGGGAVEFVRCLGKNSVDHDS----
NP_000228.1_Hsapiens_LPL              -SPGFAIQKIRVKAGETQKKVIFCSR-EKV-SHLQKGKAPAVFVKCHDKSLNKKSG----
XP_015135900.1_Ggallus_LPL            -PFAFTIQRVRVKSGETQKKVVFCSR-DGS-SRLGKGEEAAIFVKCLEQPVSRKRGGAKK
XP_002934038.1_Xtropicalis_LPL        -AYLFNIQKIRVKSGEFQKKLVFCSK-EGNFASLQRGKEPVVFVKCNEHAKKKQE-----
XP_029931044.1_Mmurdjan_Lpl1b         -DNDFMIRRMRIKAGETQAKVSFRAK-DAEFAHLVQGGDAVTFLKSKEDQNSRRRERLHR
XP_013996657.1_Ssalar_Lpl1b           -SNEFMVRRIRVKTGETQSKVIFRAESDGKFAHLVQGGDPVTFTKSKEDQNSRTHERNHR
XP_036842445.1_Omykiss_Lpl1b          -SNEFMVRRIRVKTGETQSKVIFRAESDGKFAHLVQGGDPVTFSKSKEDQNSRTHERIHR
XP_005174466.1_Drerio_Lpl1b           -SNQFTIRRMRIKSGETQAKVIFRAK-ETEFASLVQGGDPVVFVKSSETPQSRREERKHR
XP_042575005.1_Ccarpio_Lpl1b          -TNQFMIRKMRIKSGETQSKLIFRPK-EGEFVSLVQGGDAVVFVKSSETPQSRRQQRQHR
XP_026874726.2_Eelectricus_Lpl1b      -TGQFRIRRMRVKSGETQAKLMFRPK-HEDFGVLTQGGDSVIFVKSDENPHSRREAKRHR
XP_034153683.1_Phypophthalmus_Lpl1b   -TNRFLIRKLRIKSGETQAKLIFRPK-DVEFGSLTQGADGVIFVKSNDNPQSRKEERLHR
XP_017351928.1_Ipunctatus_Lpl1b       -TNQFLIRKMRIKSGETQAKVIFQPK-DVEFGSLTQGADGVIFVKSNANPQSRKEERLHR
XP_049334816.1_Amexicanus_Lpl1b       -ANHFMIRRMRVKSGESQAKVIFSPK-EVELGYLIQGGDAVVFVKSNENLQSKKEARLHR
XP_036412281.1_Cmacropomum_Lpl1b      -TNEFKIRKMRIKSGETQTKVIFRPK-DVEFGDLIQGGDSVVFVKSKENEHSKREARSHR
XP_017552653.2_Pnattereri_Lpl1b       -TNEFNIRKMRIKSGETQAKVIFRPK-DVEFEDLIQGGDAVVFVKSKEHEQSKREARSHR
XP_042561452.1_Charengus_Lpl1b        -SNEFMIRRLRIKSGETQSKLIFLSK-VGELAYLTQGGEDAIFVKSKEDQESRREARLHR
XP_030630539.1_Cchanos_Lpl1b          -ANEFMIRRLRVKSGETQAKAIFQTK-EGEFGYLKQGGDTLVFVKSKENPVSRREERLHR
XP_035276326.1_Aanguilla              -LSPFYARRLRVKAGETQARVVFGAK-DGEFVFLSRGGGYVTFIRSKEDPASLKQERLHR
XP_035276363.1_Aanguilla              -THLFNTRVLI-----QMMVCVYCID-----------AKTIF------------------
XP_028672356.1_Ecalabaricus_LPL       -GYKFNIRKIRVKAGETQAKMTFSAK-DGEMAALVRGGEFVDFVKSKEDQASKRHARRHG
XP_006640052.1_Loculatus_LPL          -SSSFMIRKLRVKAGETQAKVEFHAQ-NGDFAELKRGGDFATFVRSKESHVSKRHAREHR
XP_023674680.1_Pkingsleyae_Lpl1b      -SSSFLVRKMRIKAGETQARAVFTVK-QGDIVHLVKGGEEIVFVKSEEDEASRKQRRMHR
XP_036409940.1_Mcyprinoides_Lpl1b     -QQQFKIRRLRIKSGETQAKVVFTAK-DGEFAHLVRGGEYVSFMRSKEDHANRKQERLHR
NP_001306750.1_Csemilaevis_Lpl1a      -SSTFHIRKMRVKCGENQSRMILSSK-DDEFAFLIRGGGDAVFVMTK-DITSRREKLGHK
XP_023699829.1_Pkingsleyae_Lpl1a      -SKMFWVRKIRVKAGETQAKLVFRAK-DGEAAYLSRGGDYAVFVKSNENESSRKAERLFR
XP_012683808.2_Charengus_Lpl1a        -SSNFHIRRLRVKAGETQARVIFTSK-DGEFAYLTRGGEPTVFVKSKDHQTSRKQERLHK
XP_030640550.1_Cchanos_Lpl1a          -TNTFHIRKIRVKSGETQAKVIFSAK-EGEFAYMSQGGEPAVFVKSKEAQSSRKERKLHK
NP_571202.1_Drerio_Lpl1a              -SDTFHIRKLRIKSGETQSKIIFSAK-ESEFSYLSRGGEAAVFVKDKEAQSSRKNQRLHK
XP_042568381.1_Ccarpio                -PDTFHIRKLRVKSGETQSKIIFRAK-EGEFSYLSRGGEGRSFMKEKEAQSSRKNQRLHK
XP_018919916.2_Ccarpio_Lpl1a          -PDTFHIRKLRIKSGERQSKIIFSAK-EGEFSYLSRGGEAAVFVKEKEAQSSRKNQRLHK
XP_042568359.1_Ccarpio                -PDTFHIRKLRVKSGETQSKIIFRAK-EGEFSYLSRGGEAAVFMKEKEAQSSRKNQRLHK
XP_026861273.2_Eelectricus_Lpl1a      -ADTLHIRKLRIKSGETQSRVIFVAK-DGEFSYLTRGGDAAVFVKDKEAQSSHKSERLHK
XP_026786482.1_Phypophthalmus_Lpl1a   -PDTFHIRKLRIKCGETQSRVIFIAK-EGEFSYLARGGEAAVFVKDKEAQSSRKNERLHK
NP_001316205.1_Ipunctatus_Lpl1a       -PDTFHIRKLRVKSGETQSRVIFIAK-EGEFAYLTRGGEASMFVKDKEAQSSHKNKRLHK
XP_007240188.3_Amexicanus_Lpl1a       -PDTFHIRRLRVKSGETQSRVIFIAK-EGEFSYLARGGDAAVFVKDKEAQSSQKNAKLHK
XP_017548830.1_Pnattereri_Lpl1a       -PDTFHVRKLRVKSGERQSRVIFVAK-DGEFSYLARGGDAAVFVKDKEAQSSKKNERLHK
XP_036442904.1_Cmacropomum_Lpl1a      -PDTFHIRKLRVKSGERQSKVIFIAK-EGEFSYLARGGDAAVFVKDKEAQSSQKNERLQK
XP_018583848.2_Sformosus_Lpl1a        -SNKFWVRRLRIKSGETQAKVIFSAK-NGEFAFLSRGGDHAVFVKSKDQQSSRKEERRHK
XP_035272161.1_Aanguilla              -NKSFQIRKLRIKAGETQAKVIFSAK-EGEFAYLVRGGKEAVFVKSKEEKSSQKEERLHK
XP_036376712.1_Mcyprinoides_Lpl1a     -NNKFNIRKLRVKVGETQAKVIFSAK-EGEFEYLVRGGNEAVFVKSKEDQSSRKEERLHK
XP_030228467.1_Gmorhua_Lpl1a          -RSKFHIRKLRVKSGETQSRAIFSSK-DGEFAFMIRGGEDAVFVKTRDDNMNRQEKRMHK
```

```
XP_010869814.1_Elucius_Lpl1a        -NSNFHIRKIRIKAGETQSKGIFSAK-DGEFAYLVRGKDDAVFVKSKEDNLSRKEKTMHR
XP_014071701.1_Ssalar               -NKNFHIRKIRVKAGETQSRVIFSAK-DGEFAYLIRGKDDVVFVKSKEDNMSRKEKKMHR
NP_001118076.1_Omykiss              -NKNFHIRKMRVKAGETQSRVIFSAK-DGEYAYLIRGKDDVVFVKSKEDNMSRKEKTMHR
XP_014004153.1_Ssalar               -NSNFHIRKMRVKAGETQSKVIFSAK-DGEFAYLVRGKDDAVFVKSKEDNMSRKEKTMHR
XP_021435556.2_Omykiss              -NSNFHIRKMRVKAGETQSKVIFSAK-DGEFAYLVRGKDDAVFVKSKEDNMSRKEKTMHR
XP_003974174.2_Trubripes_Lpl1a      SSSRVHIRKLRIKSGESQSKVIFGAK-DGEYVHLVRGGEDGVFVKLKEDPMSRKEKLMHK
NP_001266682.1_Oniloticus_Lpl1a     -STKFNIRKLRIKCGETQSKVIFTAK-DGEFVDLTRGGENAVFVKSKEDNQSRKEKLANK
NP_001295924.1_Olatipes_Lpl1a       -SSKFHIRKLRIKSGETQSKVIFNAK-EGEFADLVRGGEDAVFVKSKEDNISRKERRMHR
XP_029905863.1_Mmurdjan_Lpl1a       -HSSFHIRKMRIKSGEKQSKVIFSAK-EGEFAYLIRGGEDAVFVKSREDNRSRKEKLMHK
XP_035504156.1_Smaximus_Lpl1a       -SSRFHIRKLRVKSGETQSKVIFSPK-EGEFSYLVRGGEDSVFVKSREDTVSRKDKLLQK
XP_040040562.1_Gaculeatus_Lpl1a     -SSKFHIRKLRIKSGETQSKVIFSAK-DGEFAYLVRGGEEGVFVKSKEDNMNRKEKLMHK
XP_039666916.1_Pfluviatilis_Lpl1a   -GGKFHIRKLRIKSGETQSKVIFNAK-EGEFAYLVRGGEDGVFVKSKEDQMNRKEKLMHK
XP_051233158.1_Dlabrax_Lpl1a        -SSKFHIRKLRIKSGETQSKVIFSAK-EGEFAYLVRGGEDAVFVKSKEDNRSRKEKLMHK
                                        .  . :                                *

XP_007899209.1_Cmilii_LPL           ---------------------------------------------------
XP_030219625.1_Gmorhua_Lpl1b        ---------------------------------------------------
XP_005989949.1_Lchalumnae_LPL       KH-------------------------------------------------
XP_028568270.1_Pmuralis_LPL         ---------------------------------------------------
NP_000228.1_Hsapiens_LPL            ---------------------------------------------------
XP_015135900.1_Ggallus_LPL          ASKENSAHESA----------------------------------------
XP_002934038.1_Xtropicalis_LPL      ---------------------------------------------------
XP_029931044.1_Mmurdjan_Lpl1b       RKMHGSFFKQGQNEAAIA---------------------------------
XP_013996657.1_Ssalar_Lpl1b         LKMQGSFFKQGLNGAATDTSPSSSSLPPSPEH-------------------
XP_036842445.1_Omykiss_Lpl1b        LKMQGSFFKQGLNDAATDTSSSSSPPSPEH---------------------
XP_005174466.1_Drerio_Lpl1b         LKHHGSFFKQGINEATADLRKPPAALHNNQSTETTTTANTSQVNTEHWTGI
XP_042575005.1_Ccarpio_Lpl1b        LKHHGSFFKQSINDATADMSKEPALLHNESTEIMTETTAQVNTTNALRFY-
XP_026874726.2_Eelectricus_Lpl1b    LKMHGSYFKEDMKQQV-----------------------------------
XP_034153683.1_Phypophthalmus_Lpl1b LKMHGSFFKQDGNETAADVKNPETQTENTVTATTKMVTQSS----------
XP_017351928.1_Ipunctatus_Lpl1b     LKMHGSFFKQDGTEAATDVKNPETQTENAVTATTVMVTHSS----------
XP_049334816.1_Amexicanus_Lpl1b     LKMHGSFFKEGTNE-TGDIKNEQPVTQSTNVATTEKRVETTTKKATS----
XP_036412281.1_Cmacropomum_Lpl1b    LKMHGSFFKEGVNESTTDVKKEEPVMQSSKEESTEKPMEVNTKKATTS---
XP_017552653.2_Pnattereri_Lpl1b     LKMHGSFFKEGVNESTTDVKKEESGMQSTEKPVEVTTKKATSS--------
XP_042561452.1_Charengus_Lpl1b      LKMHGSFFNQATE--------------------------------------
XP_030630539.1_Cchanos_Lpl1b        LKMHGSFFKQGMNEAEKEMKENS----------------------------
XP_035276326.1_Aanguilla            LKMIGSSFKQSAE--------------------------------------
XP_035276363.1_Aanguilla            ---------------------------------------------------
XP_028672356.1_Ecalabaricus_LPL     HRRSKNPWKSNSD--------------------------------------
XP_006640052.1_Loculatus_LPL        RRMYASPWGKKMD--------------------------------------
XP_023674680.1_Pkingsleyae_Lpl1b    LKMYGSSFKQSTM--------------------------------------
XP_036409940.1_Mcyprinoides_Lpl1b   LKMNGSLFKRSAE--------------------------------------
NP_001306750.1_Csemilaevis_Lpl1a    RRMKGTLFGQNDA--------------------------------------
XP_023699829.1_Pkingsleyae_Lpl1a    RKKQGSSFRKSEDVDSGNNSTIATQ--------------------------
XP_012683808.2_Charengus_Lpl1a      LKMNGSNFKQNTA--------------------------------------
XP_030640550.1_Cchanos_Lpl1a        LKMEGSSFKKDGE--------------------------------------
NP_571202.1_Drerio_Lpl1a            MKMHGSSFKQNNE--------------------------------------
XP_042568381.1_Ccarpio              LKMDGSSFKQSTE--------------------------------------
XP_018919916.2_Ccarpio_Lpl1a        LKMHGSSFKQSTE--------------------------------------
XP_042568359.1_Ccarpio              LKMDGSSFKQSTE--------------------------------------
XP_026861273.2_Eelectricus_Lpl1a    LRMQGSSFTKSSKE-------------------------------------
XP_026786482.1_Phypophthalmus_Lpl1a LKMHGSSFKNTALDA------------------------------------
NP_001316205.1_Ipunctatus_Lpl1a     LKMHGSSFKNTAIDA------------------------------------
XP_007240188.3_Amexicanus_Lpl1a     LKMFGSSFKKSIE--------------------------------------
XP_017548830.1_Pnattereri_Lpl1a     LKMQGSSFKKTTE--------------------------------------
XP_036442904.1_Cmacropomum_Lpl1a    LKMRGSSFKKSTE--------------------------------------
XP_018583848.2_Sformosus_Lpl1a      RKMQGSLFTKSTE--------------------------------------
XP_035272161.1_Aanguilla            LKMNGSLFKGNSE--------------------------------------
XP_036376712.1_Mcyprinoides_Lpl1a   RKMNGSLFKQNTK--------------------------------------
XP_030228467.1_Gmorhua_Lpl1a        RKMQGTLFGQNDA--------------------------------------
XP_010869814.1_Elucius_Lpl1a        LKMHGSHFTQSTE--------------------------------------
XP_014071701.1_Ssalar               LKMQGSHFKNNIA--------------------------------------
NP_001118076.1_Omykiss              LKMQGSHFKNNIA--------------------------------------
XP_014004153.1_Ssalar               LKMQGSLFKKNTA--------------------------------------
XP_021435556.2_Omykiss              LKMQGSLFKKNTA--------------------------------------
XP_003974174.2_Trubripes_Lpl1a      LKTQGSLFKSEA---------------------------------------
NP_001266682.1_Oniloticus_Lpl1a     LKTKGSLFGQQDA--------------------------------------
NP_001295924.1_Olatipes_Lpl1a       LKTEGSLFGKSDA--------------------------------------
XP_029905863.1_Mmurdjan_Lpl1a       LQMQGSSFGQNDA--------------------------------------
XP_035504156.1_Smaximus_Lpl1a       LKTQGSLFGQNDA--------------------------------------
XP_040040562.1_Gaculeatus_Lpl1a     LKMQGSLFGQNDA--------------------------------------
XP_039666916.1_Pfluviatilis_Lpl1a   LKMRGSLFGLNDA--------------------------------------
XP_051233158.1_Dlabrax_Lpl1a        LKVQGSLFGQNDA--------------------------------------
```

Figure S3. CLUSTAL multiple sequence alignment of lipoprotein lipase 2 from tambaqui with vertebrate sequences. Expanded alignment of the deduced amino acid sequences of the lipoprotein lipase 2a gene from tambaqui with 32 vertebrate sequences. * indicates identical amino acid residues among multiple sequences. Gaps introduced to optimize alignment are represented by (-). Highlighted colors indicate domains previously described in human LPL. Alignment was performed using MUSCLE (3.8).

```
XP_007899210.1_Cmilii_LPL2            MRLCLCAKSI-LLFLSLSLLPASAHE-ANTN--TNTTDTVPRTQGNSI----------ED
XP_023674674.1_kingsleyae_Lpl2b       MKVEGYCCIL-LIVFNTAVPPVKLLNEDE----SNFFGNVLEPL-KDLFNQESQTEDSNH
XP_014340461.1_Lchalumnae_LPL2        --MKPITLLLLLTLPVLIPATQPSE-NDLN--SQHFDAEPEEF----------------
XP_028672354.1_Ecalabaricus_LPL2      MKSDTLSRLL-LFLFSCQLLCATTVE-EVAS--ATSAGSPTTAE-AKLALP-------DN
XP_006640053.2_Loculatus_LPL2         MKKQKCCYFL-VFLLGVTVFPTSSSMRADV---LGLLDNSLEPQ-EHAADA---------
XP_026861314.2_Eelectricus_Lpl2a      MKIWPINYLF-LLISGFTVICVTSVE-EELS--DTFHVNFLEPL-KNLSPQ-------RV
XP_026786600.2_Phypophthalmus_Lpl2a   MNIRPFKCVF-------CVFWLAALE--GLSCTLSLHDNFLEPL-KSLGLQ-------RE
XP_007240187.1_Amexicanus_Lpl2a       MRFWPLEILV-LVVTGSSVLCESSLE-GELP--TSLRDNFLEPISWSLFHQ-------RE
XP_017548831.1_Pnattereri_Lpl2a       MRIWPFTSLV-LLVSASTVVCGTSLE-GELS--VSLRDNFLEPL-RSLFRQ-------RE
XP_036442371.1_Cmacropomum_Lpl2a      MRFWPLTSLV-LLVLGSTVLCGTSLE-GELS--VSLRDNFLEPL-RSLLRQ-------RE
XP_023699905.1_Pkingsleyae_Lpl2a      MKTRGYQVI--LLVLCAHFISVTALE-EEIS--DSAFDNFLELI-PSLRKE-------RS
XP_002666287.3_Drerio_Lpl2a           MNIRSLLCVFLWLVLNITGPFIAALEEGNVL--NGVFDHFLEDL-RDL----------SD
XP_042604741.1_Ccarpio_Lpl2a          MNILSFQCVS-WLVLSFTGLFVAALEKEIFS--NELFDNFLEDL-RDL----------SD
XP_018583841.2_Sformosus_Lpl2a        MRSAGYHRLCSTLVLSSAFLFVTPSE-EDISSASSLFGNILEPI-WGF------------
XP_019904837.1_Elucius_Lpl2a          MKGLHVNCFY-VFLLN-AVFYVTTL--GEHS--VSINDNFMDNF-KDLIEN-------ND
XP_014071700.2_Ssalar                 MKGLQVHCLY-FLVLD-AVFYVASLE-EGDS--ISTLDNLMDNF-KDLIRN-------ND
XP_021459327.2_Omykiss                MKGLQVHCLY-FLMLN-VVFYVASLE-EGDS--ISTLDNFMDNF-KDLMRN-------ND
XP_045553204.1_Ssalar                 -----MCCVH----------------------IPLLDNLMDNF-KDLFQN-------ND
XP_021435376.2_Omykiss                MKGLQVQCLY-FLVLN-SVLSVSFLE-EDHS--VHTLDNLMDNF-KDLFQK-------ND
XP_004068405.1_Olatipes_Lpl2a         MRAWQLGFLF-VVVSNAAVQHVMSLEGEELA--DSTFGNFLDPL-KDLFEP-------ED
XP_040040767.1_Gaculeatus_Lpl2a       MKVWRVRLLY-FLALNAAVQHVTSLE-EKRT--GSIFDNLLDPL-TELFEH-------KD
NP_001292532.1_Trubripes_Lpl2a        MKAWKSGVVF-ILVLNAAVCHVTTLE-GELA--DSISGNFLDPI-KDLLND-------RD
XP_030228828.1_Gmorhua_Lpl2a          MKGWPVRCLY-FLVLNAAVQSVTSLE-EELS--DSIFGNFLDPL-KDLFEP-------RE
XP_008333524.1_Csemilaevis_Lpl2a      MKAWRIQFLC-FLVFGAAVQYVTSVE-EELA--DSIFGNFLDPL-NDLFQH-------KD
XP_029905846.1_Mmurdjan_Lpl2a_Lpl2a   MKGWQIRCLF-FLVLYTAVENVTSLE-EELA--DSIFGNFLDPL-KDLFDQ-------KD
XP_003439890.1_Oniloticus_Lpl2a       MRAWKVRFLC-FVVLNAAVQNVTSLE-EELP--DSIFGNFLDPV-KAFFDH-------KA
XP_035504157.2_Smaximus_Lpl2a         MKAWRLGFLY-VLVLIAAVQHVTSLE-EELA--DSIFGNFLEPL-NDLFDH-------KG
XP_039667734.1_Pfluviatilis_Lpl2a     MKAWRLRFLY-FLVLNAAVQYVTSLE-EELS--DSIFGNFLDPL-KDLFDH-------KD
XP_051233284.1_Dlabrax_Lpl2a          MKAWQAGFLY-FVVLNAAVQYVTSLE-EELT--DSIFGNFLDPL-KDLFDR-------KD
XP_030639366.1_Cchanos_Lpl2a          MKILSYQCHL-LLLLSSTVLCVSTLE-EELS--NTIFDNFLEPL-NDLFHR-------KD
XP_012683807.1_Charengus_Lpl2a        MKLRFHQCLF-WLVLSCTVLVVSSLE-DVLS--QSNFDNFLDPL-KDLFTL-------KD
XP_035270726.1_Aanguilla_Lpl2a        MKRGAYQYLF-LFVTSLSVFPVTPLE-EELS--NSLFDNFLEPI-KGLFNK-------KD
XP_036374600.1_Mcyprinoides_Lpl2a     MKRGAYQCLF-LFVICLTVFPVTPLE-EELS--DTLFDNFLEPL-KGLFNK-------ND


XP_007899210.1_Cmilii_LPL2            --LTSVRTRFSLRVPSQPDEDVCHVIPGRAESLAECGFNATSKSFLIIHGWSV-------
XP_023674674.1_kingsleyae_Lpl2b       TEANPTHTKFSVRSPGPPHEDECYIEAGNAASLHKCRFNATSRTFLVIHGWTV-------
XP_014340461.1_Lchalumnae_LPL2        ---NSVHTKFSLRTPLDPEEDVCYIVPGRADTLNKCNFNTTTKTFLVIHGWTV-------
XP_028672354.1_Ecalabaricus_LPL2      --SKSILTRFSLRQPSEPEEDVCYIIPGNPDSLHSCNFNLTSKTFIVIHGWTV-------
XP_006640053.2_Loculatus_LPL2         --SDSALTRFSLRKPSDPEDDVCYVGPSKPDSLSACSFNHTSKTFLIIHGWTV-------
XP_026861314.2_Eelectricus_Lpl2a      -KVRGVHAKFSLRKPALPDDDMCYIVPGKEESLGKCSFNTTAKTFLVIHGWTV-------
XP_026786600.2_Phypophthalmus_Lpl2a   --AGEAHSKFSLRKPSLPDDDICYIVPGKTETLHNCNFNSTAKTFLVIHGWTV-------
XP_007240187.1_Amexicanus_Lpl2a       --ANNVHAKFSLRKPSLPDDDICYIVPGEEETLGNCNFNNTAKTFLVIHGWTV-------
XP_017548831.1_Pnattereri_Lpl2a       --ASNVLAKFSLRRPSLPDDDVCYIVPGKEETLGNCNFNSTAKTFLVIHGWTV-------
XP_036442371.1_Cmacropomum_Lpl2a      --ASDVLAKFSLRRPSLPDDDVCYIVPDKEETLGNCNFNSTAKTFLVIHGWTV-------
XP_023699905.1_Pkingsleyae_Lpl2a      ---GSVSARFSLRKPSDPDDDVCYIVTGDPESLKECDFNTTSKTFLVIHGWTV-------
XP_002666287.3_Drerio_Lpl2a           --VKKLNVKFSLRNPSQPDDDVCYIVRGKAETLSSCNFNHTSKTILVIHGWTV-------
XP_042604741.1_Ccarpio_Lpl2a          --VNKANVKFSLRSPSHPDEDVCYIVHGKPETLNSCSFKNTSKTFMVIHGWTV-------
XP_018583841.2_Sformosus_Lpl2a        ---KTAHAKFSLRRPLDPTDDICYIVTGSLESLIKCNFNATSKTFLVIHGWTV-------
XP_019904837.1_Elucius_Lpl2a          -ANSHAYAKFSLRKPSMPDDDTCYISPGNSESLKECSFNSASKTFLVIHGWTV-------
XP_014071700.2_Ssalar                 -ATPHAYAKFSLRKPLMPEDDICYIIPGNPESLKECTFNSTSKTFLVIHGWTV-------
XP_021459327.2_Omykiss                -ATPHAYAKFSLRKPLMPEDDICYIIPGNPESLKECTFNSTSKTFLVIHGWTV-------
XP_045553204.1_Ssalar                 -ATPHAYTKFSFRKPLMPEDDICYIIPGNPESLKECTFNSTSKTFLVIHGWTV-------
XP_021435376.2_Omykiss                -ATPHAYTKFSLRKPLMPEDDICYIIPRNPESLKECTFNSTSKTFLVIHGWTV-------
XP_004068405.1_Olatipes_Lpl2a         -DKNSTIARFSLRKPSHPDDDLCFIIPGQPESLTACTFNRTSKTFLLIHGWTL-------
XP_040040767.1_Gaculeatus_Lpl2a       -AGDQPAAKFSLRKASRPDDDLCYIVPGKPDSLAACTFNSTSKTFLVIHGWTVSGAAFHH
NP_001292532.1_Trubripes_Lpl2a        -DSNQTVARFSLRKPSHPDADQCYIVPGRADTLAACTFNRTSKTFMVIHGWTL-------
XP_030228828.1_Gmorhua_Lpl2a          LESNATAVRFSLRKPSMPEDDLCYLTPGKPESLAACSFNSTSKTFLVIHGWTV-------
XP_008333524.1_Csemilaevis_Lpl2a      -GINETIAKFSLRKPSYPDDDICYIIPGNPDSLNACSFNSTSKTFLVIHGWTV-------
XP_029905846.1_Mmurdjan_Lpl2a         -DSNESIAKFSLRKPSQPDDDLCYIIPGKPESLASCTFNSTSKTFLVIHGWTI-------
XP_003439890.1_Oniloticus_Lpl2a       -ESNATVAKFSLRKPSHPDDDLCYIIPGKPDSLATCTFNSTSKTFLIIHGWTL-------
XP_035504157.2_Smaximus_Lpl2a         -DSNQTVAKFSLRKPSHPDDDLCYIVPGKPDSLAACTFNSTSRTFLVIHGWTL-------
XP_039667734.1_Pfluviatilis_Lpl2a     -DSNQSVAKFSLRKPSHPDEDLCYIVPGKPDSLAACTFNSTSKTFLVIHGWTL-------
XP_051233284.1_Dlabrax_Lpl2a          -ESNQTVAKFSLRKPSHPDDDLCYIVPGKADSLAACTFNSTSKTFLVIHGWTL-------
XP_030639366.1_Cchanos_Lpl2a          --ANKTYAKFSLRKPSQPEEDVCYIVPNEPDSLSNCAFNSTAKTFLVIHGWTV-------
XP_012683807.1_Charengus_Lpl2a        --ATGPQVKFSLRKPNQPDDDVCYIVPGKPESLSGCTFNSTSKTFLVIHGWTV-------
XP_035270726.1_Aanguilla_Lpl2a        --GSSAYAKFSLRKPSDPDEDVCYIVPGSPESLSDCQFNTTSKTFLVIHGWTV-------
XP_036374600.1_Mcyprinoides_Lpl2a     --DNSAYAKFSLRKPSDPDDDVCYIIPGKPETLSDCHFNTTAKTFLVIHGWTV-------
                                              .**.* .  *  * *.:      :*  * *: ::.::::****::


XP_007899210.1_Cmilii_LPL2            ----------------------SGMFESWIHKLVTALYQREPAANVLVVDWLNRAHQHYP
XP_023674674.1_kingsleyae_Lpl2b       ----------------------SGMLASWISKLVSALFEREPDANVIVVDWLSRAHNHYF
```

```
XP_014340461.1_Lchalumnae_LPL2          ----------------------SGMFESWISKMVAALYKREHDANVIVVDWLNLAHQHYP
XP_028672354.1_Ecalabaricus_LPL2        ----------------------SGMFESWVPKLVAALYERERDANVIVVDWLNRAHQHYS
XP_006640053.2_Loculatus_LPL2           ----------------------SGMFEGWVAKLVSALYERERDANVIVVDWLSMAHNHYP
XP_026861314.2_Eelectricus_Lpl2a        ----------------------SGLFESWVAKLVAALYSREKDANVIVVDWLNTAQNHYA
XP_026786600.2_Phypophthalmus_Lpl2a     ----------------------SGLFESWVAKLVAALYNREKDANVVVVDWLDTAQNHYG
XP_007240187.1_Amexicanus_Lpl2a         ----------------------SGLFESWVAKMVAALYGREKDANVIVVDWLNTAQDHYI
XP_017548831.1_Pnattereri_Lpl2a         ----------------------SGLFESWVAKMVAALYDREKDANVIVVDWLDMAQNHYI
XP_036442371.1_Cmacropomum_Lpl2a        ----------------------SGLFESWVAKMVAALYDREKDANVIVVDWLDAAQNHYV
XP_023699905.1_Pkingsleyae_Lpl2a        ----------------------SGLFEGWVGRLVAALYEREPSANVIVVDWLSTAQNHYI
XP_002666287.3_Drerio_Lpl2a             ----------------------SGLFESWVEKLVAALYNREKDANVIVVDWLDTAQDHYV
XP_042604741.1_Ccarpio_Lpl2a            ----------------------SGLFESWVEKLVAALYNREKDANVIVVDWLDMAQNHYV
XP_018583841.2_Sformosus_Lpl2a          ----------------------TGMFESWVGKLVAALYKREPSANVIVVDWLSTAHNHYT
XP_019904837.1_Elucius_Lpl2a            ----------------------SGLFESWVAKLVSALYEREPNANVIVVDWLYTAQNHYL
XP_014071700.2_Ssalar                   ----------------------SGLFESWVAKLVSALYKREQEANVIVVDWLYTAQNHYP
XP_021459327.2_Omykiss                  ----------------------SGLFESWVAKLVSALYKREQEANVIVVDWLYTAQNHYP
XP_045553204.1_Ssalar                   ----------------------SGLFESWVAKLVSALYEREQDANVIVVDWLYTAQNQYP
XP_021435376.2_Omykiss                  ----------------------SGLFESWVAKLVSALYEREQDANVIVVDWLHTAQNHYP
XP_004068405.1_Olatipes_Lpl2a           ----------------------SGMYGSWMPKLVSALFEKEQSANVIVVDWLSLAQNHYA
XP_040040767.1_Gaculeatus_Lpl2a         FHQEPPRCCIREFPTVFLFKQLSGMFESWVTKLVSALYAREETANVVVVDWLASAQNHYA
NP_001292532.1_Trubripes_Lpl2a          ----------------------SGMFESWVSKLVSALYEREHGANVIVVDWLTLAQNHYV
XP_030228828.1_Gmorhua_Lpl2a            ----------------------SGMFESWVSKLVSALYRREHTANVIVVDWLGTAQNHYI
XP_008333524.1_Csemilaevis_Lpl2a        ----------------------SGMFESWVSQLVEALYNREQTANVIVVDWLSSAQNHYV
XP_029905846.1_Mmurdjan_Lpl2a           ----------------------SGMFESWVAKLVSALYERERTANVIVVDWLSSAQNHYV
XP_003439890.1_Oniloticus_Lpl2a         ----------------------SGMFESWMPKLVSALYEREQTANVIVVDWLNSAQNHYV
XP_035504157.2_Smaximus_Lpl2a           ----------------------SGMFESWVAKLVLALYEREQTANVIVVDWLTSAQNHYV
XP_039667734.1_Pfluviatilis_Lpl2a       ----------------------SGMFQSWVAKLVSALYDREQTANVIVVDWLTSAQNHYV
XP_051233284.1_Dlabrax_Lpl2a            ----------------------SGLFESWMAKLVSALYEREQTANVIVVDWLTSAQNHYV
XP_030639366.1_Cchanos_Lpl2a            ----------------------IGLFESWVAKLVAALYERERDANVIVVDWLDVAQNHYV
XP_012683807.1_Charengus_Lpl2a          ----------------------SGLFESWVAKLVAALFDREQSANVIVVDWLSTAQNHYV
XP_035270726.1_Aanguilla_Lpl2a          ----------------------SGMFESWVAKLVAALYEREHSANVIVVDWLNTAQNHYI
XP_036374600.1_Mcyprinoides_Lpl2a       ----------------------SGMFESWVAKLVAALYERERDANVIVVDWLSTAQNHYV
                                                               *:  .*: .:* **: .*  ***:*****  *:::* 

XP_007899210.1_Cmilii_LPL2              IAAQNTVLVGEDIAKFLNWIKELVNVTLDNVHLIGYSLGAHVAGFAGSHVANKIGRITGL
XP_023674674.1_kingsleyae_Lpl2b         TSAQDTELVGQDVARLIDWIMDKTGVSAENLHLIGYSLGAHVAGFAGSNTSRKVGRITGL
XP_014340461.1_Lchalumnae_LPL2          IAAQNTKLVGQDIAQFIDWIKEKINFPLDNLHLIGYSLGAHVAGFAGSHTANRVGRITGL
XP_028672354.1_Ecalabaricus_LPL2        VAAENTKLVGCDIAWLIDWMEETANIPLQNVHLIGYSLGAHVAGFAGSHTTNKVGRITGL
XP_006640053.2_Loculatus_LPL2           IAAKKTKLVGQDIGRFLDWLEEEVNFPLDKVHLIGYSLGAHVAGFAGSHASSKVGRITGL
XP_026861314.2_Eelectricus_Lpl2a        LAAQSTQKVGQEVAHFIDWIEEMTNIPLDKLHLIGYNLGAHVAGFAGSYANNKVGRITGL
XP_026786600.2_Phypophthalmus_Lpl2a     VAAQNTQEVGQEVGRFIDWIEETTNIPLEKIHLIGYSLGAHVAGIAGSHASRKVGRITGL
XP_007240187.1_Amexicanus_Lpl2a         VAAQNTQKVGQEVGRFIDWMEDKANIPLEKLHLIGYSLGAHVAGFAGSHATNKVGRITGL
XP_017548831.1_Pnattereri_Lpl2a         VAAENTQKVGQEVGHFIDWIEEATNIPLEKVHLIGYSLGAHVAGFAGSHTTNKVGRITGL
XP_036442371.1_Cmacropomum_Lpl2a        VAAENTEKVGQEVGHFIDWIEGATNIPLEKVHLIGYSLGAHVAGFAGSHATNKVGRITGL
XP_023699905.1_Pkingsleyae_Lpl2a        VAAHNTKMVGQNIANFINWMEKTTHLSLDHLHLIGYSLGAHVAGFAGNHVSNKIGRITGL
XP_002666287.3_Drerio_Lpl2a             VAAQNTKMVGREIGLFIDWIEETSNVPLENLHLIGYSLGAHVAGFAGSHTTNKIGRITGL
XP_042604741.1_Ccarpio_Lpl2a            VAAHNTKMVGQEIGLFIDWLEETARIPLEKLHLIGYSLGAHVAGFAGNHATNKVGRITGL
XP_018583841.2_Sformosus_Lpl2a          VAAQNTKRVGENVAHFITWMKEATGLSLESLHLIGYSLGAHVAGFAGSHVSNKIGRITGL
XP_019904837.1_Elucius_Lpl2a            VAAQNTKMVGQEIAHFIDWLEEETNILLENLHLIGYSLGAHVAGFAGSHTLNILGRITGL
XP_014071700.2_Ssalar                   VAAQNTKMVGQEIARFIDWLEEATNIPLENLHLIGYSLGAHVAGFAGSHASNKVGRITGL
XP_021459327.2_Omykiss                  VAAQNTKMVGQEIARFIDWLEEATNIPLENLHLIGYSLGAHVAGFAGSHASNKVGRITGL
XP_045553204.1_Ssalar                   VAAQNTKMVGQEIAHFIDWLEEATNIPLENLHLIGYSLGAHVAGFAGSHASNKVGRITGL
XP_021435376.2_Omykiss                  VAAQDTKMVGQEIAHFIDWLEEATNIPLDNLHLIGYSLGAHVAGFAGSHASNKVGRITGL
XP_004068405.1_Olatipes_Lpl2a           VAAQNTKAVGQEIARFIDWIEETTNIPLDNIHLIGYSLGAHVAGFAGSHAANKVGRITGL
XP_040040767.1_Gaculeatus_Lpl2a         VAAQNTREVGREVARFIDWIEETTNMPLENLHLIGYSLGAHVAGFAGSHATNKVGRITGL
NP_001292532.1_Trubripes_Lpl2a          LAAQKTKAVGQEIARFIDWIEESTNAPVENIHLIGYSLGAHVAGFAGSHVTNKVGRITGL
XP_030228828.1_Gmorhua_Lpl2a            VAAQSTQLVGQEIAGFIDWIEETTNMPLENLHLIGYSLGAHVAGFAGSHSTNKVGRITGL
XP_008333524.1_Csemilaevis_Lpl2a        IAAQKTKAVGREIARFIDWIEETTHLPLENIHLIGYSLGAHVAGFAGSHATNKVGRITGL
XP_029905846.1_Mmurdjan_Lpl2a           VAAQKTKAVGQEIARFIDWIEETTNIPLENLHLIGYSLGAHVAGFAGSHATNKVGRITGL
XP_003439890.1_Oniloticus_Lpl2a         VAAQKTKAVGHEIAQFIDWIEETTNMPLENIHLIGYSLGAHVAGFAGSHATNKVGRITGL
XP_035504157.2_Smaximus_Lpl2a           VAAQKTKAVGQEIARFIDWLEETTNMPLENIHLIGYSLGAHVAGFAGSHATNKVGRITGL
XP_039667734.1_Pfluviatilis_Lpl2a       VAAQNTKTVGHEIARFIDWIEETTNMPLENIHLIGYSLGAHVAGFAGSHATNKVGRITGL
XP_051233284.1_Dlabrax_Lpl2a            VAAQNTKAVGQEIARFIDWIEETTNMPLENIHLIGYSLGAHVAGFAGSHATNKVGRITGL
XP_030639366.1_Cchanos_Lpl2a            VAAQHTQKVGQEIGRFIDWIEETTNVPLENVHLIGYSLGAHVAGFAGSHTTNKVGRITGL
XP_012683807.1_Charengus_Lpl2a          IAAQNTKSVGQDIAHFIDWMEETTNIPLENLHLIGYSLGAHVAGFAGSHTSNKVGRITGL
XP_035270726.1_Aanguilla_Lpl2a          VAAQNTKMVGHDVAHFIDWIQETTNVTLENLHLIGYSLGAHVAGFAGSHASNKVGRITGL
XP_036374600.1_Mcyprinoides_Lpl2a       VAAQNTKAVGQDVAHFIDWIEENTNVPLKNLHLIGYSLGAHVAGFAGSHASNKIGRITGL
                                         :*  *  ** ::. :: *:        . :*****.*******:**.     :******

XP_007899210.1_Cmilii_LPL2              DPAGPVFEGVHAHSRLSPDDAAFVDVLHTFTRESLGMSIGIRQPIGHVDIYPNGGSFQPG
XP_023674674.1_kingsleyae_Lpl2b         DPAGPVFEGAHADRRLSPDDAHFVDVVHTFTRASLGLSIGIQRPVGHLDVYPNGGTSQPG
XP_014340461.1_Lchalumnae_LPL2          DPAGPVFEQAHAHARLSPDDAAFVDVLHTFTRGSLGLSIGIQQPVGHVDIYPNGGSFQPG
XP_028672354.1_Ecalabaricus_LPL2        DPAGPVFEGAHAHGRLSPDDAHFVDVLHTFTRGYLGLSIGIQQPVGHVDIYPNGGSFQPG
XP_006640053.2_Loculatus_LPL2           DPAGPNFEGAQAHGRLSPDDAHFVDVLHTFTRGSLGLSIGIQQPVGHVDIYPNGGISQPG
XP_026861314.2_Eelectricus_Lpl2a        DPAGPDFEGVHAHQRLSPDDAVFVDVLHTFSRGSLGLSIGIEQPVGHVDIYPNGGSFQPG
XP_026786600.2_Phypophthalmus_Lpl2a     DPAGPDFEGVHVHRRLSPDDAHFVDILHTFSGGTLGLSIGIEQPIGHVDIYPNGGSFQPG
XP_007240187.1_Amexicanus_Lpl2a         DPAGPDFEGVHAHERLSPDDAHFVDVLHTFSRGSLGLSIGIEQPIGHVDIYPNGGSFQPG
XP_017548831.1_Pnattereri_Lpl2a         DPAGPDFEGAHAHRRLSPDDARFVDVLHTYSRGTLGLSIGIEQPIGHVDIYPNGGSYQPG
XP_036442371.1_Cmacropomum_Lpl2a        DPAGPDFEGAHAHRRLSPDDAHFVDVLHTFSRGTLGLSIGIEQPIGHVDIYPNGGSFQPG
XP_023699905.1_Pkingsleyae_Lpl2a        DPAGPDFEGAHAHQRLSPDDAHFVDVLHTFNRGSLGLSIGTQQPVGHVDFYPNGGSFQPG
XP_002666287.3_Drerio_Lpl2a             DPAGPDFEGVHAHGRLSPDDAHFVDVLHTFTRGSLGLSIGIEQPVGHVDIYPNGGSFQPG
XP_042604741.1_Ccarpio_Lpl2a            DPAGPDFEGVHAHGRLSPDDAHFVDVLHTFTRGSLGLSIGIKQPVGHVDIYPNGGSFQPG
XP_018583841.2_Sformosus_Lpl2a          DPAGPDFEGENAHRRLSPDDAHFVDVLHTFSRGSLGLSIGIQQPVGHVDIYPNGGRFQPG
XP_019904837.1_Elucius_Lpl2a            DPAGPDFEGEHAHRRLSPDDAHFVDVLHTFSQGSLGLSIGIQQPVGHVDIYPNGGNFQPG
XP_014071700.2_Ssalar                   DPAGPDFEGEHAHRRLSPDDAHFVDVLHTFTRGSLGLSIGIQQPVGHVDIYPNGGSFQPG
XP_021459327.2_Omykiss                  DPAGPDFEGEHAHRRLSPDDAHFVDVLHTFTRGSLGLSIGIQQPVGHVDIYPNGGSYQPG
XP_045553204.1_Ssalar                   DPAGPDFEGEHAHHRLSPDDAHFVDVLHTFTQGSLGFSIGIQQPVGHVDIYPNGGSFQPG
```

```
XP_021435376.2_Omykiss               DPAGPDFEGEHAHHRLSPDDAHFVDVLHTFTRGSLGFSSGIQQPVGHVDIYPNGGSFQPG
XP_004068405.1_Olatipes_Lpl2a        DPAGPDFEGEHAHRRLSPDDAHFVDVLHTFTRGSLGFSIGIQQPVGHVDIYPNGGHFQPG
XP_040040767.1_Gaculeatus_Lpl2a      DPAGPDFEGKHAHRRLSPDDARFVDVLHTFTRRSLGLSIGIRQPVGHIDIYPNGGNFQPG
NP_001292532.1_Trubripes_Lpl2a       DPAGPDFEGMHAHRRLSPDDAHFVDVLHTFTRGSLGLSIGIQQPVGHVDIYPNGGSFQPG
XP_030228828.1_Gmorhua_Lpl2a         DPAGPDFEGQHAHRRLSPDDAHFVDVLHTFTRGSLGLSIGIQQPVGHVDIYPNGGGFQPG
XP_008333524.1_Csemilaevis_Lpl2a     DPAGPDFEGEHEHKRLSPDDAYFVDVLHTFTRGSLGLSIGIQQPVGHVDIYPNGGSFQPG
XP_029905846.1_Mmurdjan_Lpl2a        DPAGPDFEGEHAHRRLSPDDAHFVDVLHTFTRGSLGLSIGIQQPVGHVDIYPNGGSFQPG
XP_003439890.1_Oniloticus_Lpl2a      DPAGPDFEGKHAHRRLSPDDAHFVDVLHTFTRGSLGLSIGIQQPVGHVDIYPNGGSFQPG
XP_035504157.2_Smaximus_Lpl2a        DPAGPDFEGEHAHRRLSPDDAYFVDVLHTFTRGSLGLSIGIQQPVGHVDIYPNGGSFQPG
XP_039667734.1_Pfluviatilis_Lpl2a    DPAGPDFEGEHAHRRLSPDDAHFVDVLHTFTRGSLGLSIGIQQPVGHVDIYPNGGSFQPG
XP_051233284.1_Dlabrax_Lpl2a         DPAGPDFEGMHAHRRLSPDDAHFVDVLHTFTRGSLGLSIGIQQPVGHVDIYPNGGSFQPG
XP_030639366.1_Cchanos_Lpl2a         DPAGPDFEGVHARRRLSPDDANFVDVLHTFTRGSLGFSIGIQQPVGHVDIYPNGGSFQPG
XP_012683807.1_Charengus_Lpl2a       DPAGPDFEGVHAHRRLSPDDAHFVDVLHTFTRGSLGLSIGIQQPVGHVDIYPNGGAFQPG
XP_035270726.1_Aanguilla_Lpl2a       DPAGPDFEGAHAHHRLSPDDAHFVDVLHTFTRGSLGLSIGIQQPVGHVDIYPNGGSFQPG
XP_036374600.1_Mcyprinoides_Lpl2a    DPAGPDFEGAHAHHRLSPDDAHFVDVLHTFTRGSLGLSIGIQQPVGHVDIYPNGGSFQPG
                                     ***** **  :   ******* ***::**:.   **:* *  .*:**:*.*****  ***

XP_007899210.1_Cmilii_LPL2           CDLQKAVNNIATYGIY----AFSEAVKCEHERSIHLFIDSLLYMDQPCTAYRCSSHSAFE
XP_023674674.1_kingsleyae_Lpl2b      CDLRRALEVVAQFSLADWPSAMSVAVQCEHERSVHLFIDSLLNGDTVGQAYRCGTSDMFD
XP_014340461.1_Lchalumnae_LPL2       CNLRGALESIAKHGIF----VFSEAVKCEHERSVHLFIDSLLHDEQQSMAFRCSSKQRFE
XP_028672354.1_Ecalabaricus_LPL2     CNLRGALESISTYGMF----GFSEAVKCEHERSIHLFIDSLLHEQEPSLAYRCSSKELFD
XP_006640053.2_Loculatus_LPL2        CSLRRALEKITANGLF----GVSDAVKCQHERSIHLFIDSLLHEREAAQAYRCSSADVFN
XP_026861314.2_Eelectricus_Lpl2a     CNLQGALKKVANLGLL----AMNEAIRCEHERSVHLFIDSLLNEEEASKAYRCASSDMFN
XP_026786600.2_Phypophthalmus_Lpl2a  CNLRGALENIAHYGLL----AMNEVIKCEQERSVHLFIDSLLNGAEPSKAYSCGSSTMFE
XP_007240187.1_Amexicanus_Lpl2a      CNLRGALEKLANLGLI----AMNEAIKCEHERSVHLFIDSLLNGEEPSKAYTCGSSDMFE
XP_017548831.1_Pnattereri_Lpl2a      CNLRGALEKIAHFGLV----AMNEAIKCEHERSVHLFIDSLLNGEELSKTYSCASSDMFE
XP_036442371.1_Cmacropomum_Lpl2a     CNLRGALEKIARFGLV----AMNEAIKCEHERSVHLFIDSLLNGEEPSKAYSCGSSDMFE
XP_023699905.1_Pkingsleyae_Lpl2a     CNLRGALEKIAKFGIF----AINDAVKCEHERSVHLFIDSLLHKQEASQAYFCGSPDMFE
XP_002666287.3_Drerio_Lpl2a          CNLRGALEKMASYGIF----AINNAIRCEHERSIHLFIDSLLNEEAAGRAYSCGSNDMFD
XP_042604741.1_Ccarpio_Lpl2a         CNMRGALEKIASYGIF----ALNNAIRCEHERSVHLFIDSLLNEEEASRAYSCGSNDMFD
XP_018583841.2_Sformosus_Lpl2a       CNLRGALEKIAKFGIF----AVSDAIKCEHERSVHLFIDSLVNEREASKAYSCGSSEMFD
XP_019904837.1_Elucius_Lpl2a         CSRQSTLETISKLGLF----AVTDSPKCSHERSIHLFIDSLLNEQQASKAYRCRSSDMFN
XP_014071700.2_Ssalar                CNLQSPLETISKLGLF----AINDVPRCSHERSIHLFIDSLVNEQEASMAYRCGSKEMFD
XP_021459327.2_Omykiss               CNLQSPLETISKLGLF----AINDVPRCSHERSIHLFIDSLVNEQEASMAYRCGSNDMFD
XP_045553204.1_Ssalar                CNLQSTLENISKYGLF----AITDIPRCSHERSIHLFIDSLVNEQEASMAYRCGSNDMFD
XP_021435376.2_Omykiss               CNLQSTLETISKYGLF----AITDIPRCSHERSIHLFIDSLVNEQEASMAYRCGSNDMFD
XP_004068405.1_Olatipes_Lpl2a        CNLRGALEKIANFGIL----AVTDAVKCEHERSIHLFIDSLLNEQDAVTAYRCGSSDTFD
XP_040040767.1_Gaculeatus_Lpl2a      CNLRGALEKIANLGIF----AVTDAVKCEHERSVHLFIDSLLNERETAVAYRCGSSDMFD
NP_001292532.1_Trubripes_Lpl2a       CNLRGTLEKIANFGIF----AITDAVKCEHERSVHLFIDSLLNEQDAAQAYRCSSSQTFN
XP_030228828.1_Gmorhua_Lpl2a         CNLHGALEKIANYGLF----AITDAVKCEHERSIHLFIDSLLNEQTASKAYRCGSNQMFD
XP_008333524.1_Csemilaevis_Lpl2a     CNLRGALEKIANFGLF----AITDAVKCEHERSVHLFIDSLLNKQEPAKAYRCGSNDIFN
XP_029905846.1_Mmurdjan_Lpl2a        CNLRGALEKIANLGIF----AITDAVKCEHERSVHLFIDSLLNEQEAAKAYRCGSTDMFD
XP_003439890.1_Oniloticus_Lpl2a      CNLRGALEKIANFGIF----AVSDAVKCEHERSVHLFIDSLLNEQESAKAYRCGSNDMFD
XP_035504157.2_Smaximus_Lpl2a        CNLRGALEKIANFGIF----AITNAVKCEHERSVHLFIDSLLNKEDGAKAYRCGSSDTFD
XP_039667734.1_Pfluviatilis_Lpl2a    CNLRGALEKIANFGLF----AITDAVKCEHERSVHLFIDSLLNEQEASKAYRCGSNDMFD
XP_051233284.1_Dlabrax_Lpl2a         CNLRGALEKIANFGIY----AITDAVKCEHERSVHLFIDSLLNEQEAAKAYRCGSSDMFN
XP_030639366.1_Cchanos_Lpl2a         CNLRAALEKIANFGIL----AMNDAIKCEHERSIHLFIDSLLNEQDASKAYSCGSNDMFN
XP_012683807.1_Charengus_Lpl2a       CNLRGALENIANFGIL----AMNDAVKCEHERSIHLFIDSLLNEQEAIKAYSCGSSEMFD
XP_035270726.1_Aanguilla_Lpl2a       CNLRGALEKIANFGIL----AMTDAIKCEHERSIHLFIDSLLNEQEASQAYSCGSSDMFD
XP_036374600.1_Mcyprinoides_Lpl2a    CNLRGALEKIANFGIL----AMSDAIKCEHERSVHLFIDSLLNEQEASKAYSCGSNDMFD
                                     *. . .:: ::  .:       ..   .*.:***:*******:       :: * :   *:

XP_007899210.1_Cmilii_LPL2           KGMCLNCRKNRCNTLGYNANPVRSVRHNRMYLKTRADMPFRVYHYQLKIHFSSKINHSDI
XP_023674674.1_kingsleyae_Lpl2b      RGICLSCNKGRCSRVGYDARRVRHARDHRMFTRTRSSMPFRVYHYQLKIHFSCKRTCPEL
XP_014340461.1_Lchalumnae_LPL2       KGFCLRCRKNRCNTLGYGVNKVRSKRSTKMYLKTRAHMPFRVYHYQLKIHFTSKHDHTEI
XP_028672354.1_Ecalabaricus_LPL2     HGFCLSCRKHRCNTVGYDVQPVRNRRSTKMFTRTRAIMPFRVYHYQLKIQFSNTSDHAEV
XP_006640053.2_Loculatus_LPL2        RGFCLSCRRNRCNAVGYSASRVRHRRSTKMYTRTRADMPFRVYHYQLKIHLLNTAKRSEI
XP_026861314.2_Eelectricus_Lpl2a     RGMCLKCRKDRCNTVGYDARRIRRARSIKMFTKTRAAMPFRVHHYQLKIRFQVQTNQSET
XP_026786600.2_Phypophthalmus_Lpl2a  RGVCLRCHKNRCNTVGYDARRVRKARSVKMFTKTRASMPFRVHHYQLKIHFTAQTNQSVT
XP_007240187.1_Amexicanus_Lpl2a      RGMCLSCRKNRCSIMGYDMKKVRKARSVKMFTKTRASMPFRVHHYQLKIHFTGQKKQTEI
XP_017548831.1_Pnattereri_Lpl2a      RGMCLRCHRNRCNTMGYDIKKVRKARSVKMFTKTRATMPFRVHHYQLKIHFTAQTDQTQT
XP_036442371.1_Cmacropomum_Lpl2a     RGMCLRCRKNRCNTMGYDIKKVRKARSVKMFTKTRATMPFRVHHYQLKIHFTAQPDQTQI
XP_023699905.1_Pkingsleyae_Lpl2a     RGACLSCRKSRCSTMGYDVGGARQARSATMFTKTRASMPFRVYHYQLKIHFSSNLNCSGV
XP_002666287.3_Drerio_Lpl2a          RGVCLQCRKNGCNTVGYDISKVRKARSVKMFTKTRGSMPFRVFHYQLKINFVGQINHLEM
XP_042604741.1_Ccarpio_Lpl2a         RGMCLQCRKNRCNTVGYDVSKVRKARSIKMFTKTRGSMPFRVFHYQLKINFLGKINQLAM
XP_018583841.2_Sformosus_Lpl2a       RGVCLSCHNNRCNTVGYGITKTRKARSAKMFTKTRASMPFRVYHYQLKIRFSSTVNHSEL
XP_019904837.1_Elucius_Lpl2a         RGMCLRCHRNHCNTVGYDISKVRKTRSVTMYTKTRASMPFRAFHYQLKVHFFGKVDRPDM
XP_014071700.2_Ssalar                RGMCLSCRRNHCNTVGYDISKVRKTRSVKLYTKTRASMPFRVYHYQVKIHFSSKVNRSEM
XP_021459327.2_Omykiss               RGMCLRCRKNHCNTVGYDISKVRKTRSVKLYTKTRASMPFRVYHYQVKIHFFSKVNRSEM
XP_045553204.1_Ssalar                RGMCLSCRKNHCNTVGYNIRKIRMTRSIKMYTKTKDSMPFRVYHYQLKILFSSKVDRSEM
XP_021435376.2_Omykiss               RGMCLSCRKNHCNTVGYNIRKIRMTRSIKMYTKTKDSMPFRVYHYQLKIHFSSKVDRSEM
XP_004068405.1_Olatipes_Lpl2a        RGMCLNCRKNRCNTVGYGVSKVRRARSVQMYTKTRSSMPFRVYHYQLKIHFSSKVNTSDV
XP_040040767.1_Gaculeatus_Lpl2a      RGVCLCCRKRRCNAVGYGVSRVRQAGSVQMYTRTRASMPFRVYHYQLKIHFSGQVKRSEM
NP_001292532.1_Trubripes_Lpl2a       RGMCLSCRKSRCNTVGYDISKVRKARNVQMYTKTRATMPFRVYHYQLKIHFSSKVNRSEM
XP_030228828.1_Gmorhua_Lpl2a         RGVCLSCRKSRCNTVGYDISKVRKARNVQMYTKTRASMPFRVYHYQLKIHFSSKVNRSDI
XP_008333524.1_Csemilaevis_Lpl2a     RGMCLSCRKGRCNTVGYDISKVRKARNVQMYTRTRASMPFRVYHYQLKIHFSSKVNRSEM
XP_029905846.1_Mmurdjan_Lpl2a        RGMCLSCRKSRCNTVGYDISKVRKARNVQMYTKTRASMPFRVYHYQLKIHFSSKVNRSEM
XP_003439890.1_Oniloticus_Lpl2a      RGMCLSCRKSRCNTVGYDISKVRKPRNVQMYTKTRSSMPFRVYHYQLKIHFSSKVNSSNM
XP_035504157.2_Smaximus_Lpl2a        RGMCLSCRKNRCNTVGYDISKVRKARNVQMYTKTRASMPFRVYHYQLKIHFSSKVNRSEM
XP_039667734.1_Pfluviatilis_Lpl2a    RGMCLSCRKSRCNAVGYDISKVRKARNVQMYTKTRASMPFRVYHYQLKIHFSSKVNRSEM
XP_051233284.1_Dlabrax_Lpl2a         RGMCLSCRKSRCNTVGYDISKVRKARNVQMYTKTRASMPFRVYHYQLKIHFSSKVNRSEM
XP_030639366.1_Cchanos_Lpl2a         RGMCLSCRKNRCNTVGYDVTKVRKPRSVKMFTKTRSSMPFRVYHYQLKIHVSSKMNRTEM
XP_012683807.1_Charengus_Lpl2a       RGMCLGCQKKRCNTIGYDVSKVRQARSVKMFTKTRASMPFRVYHYQLKIHFSSKVNQSEM
XP_035270726.1_Aanguilla_Lpl2a       RGVCLSCKKNRCSTVGYDVSRVRKARSAKMFTKTRASMPFRVYHYQLKMHFSSKVNQSEM
XP_036374600.1_Mcyprinoides_Lpl2a    RGVCLSCRKNRCNTVGYDVSKVRKARSVKMFTKTRASMPFRVYHYQLKIHFSCKVNQSEM
                                     .* ** *..  *. :**.    *      :: .*.  ****..***:*: .
```

```
XP_007899210.1_Cmilii_LPL2             NPRLSVSLYGTKDEVENLHIDI-KEKFAPNKTHSFLLVTETDIGDLLLIKFKWET-SSSW
XP_023674674.1_kingsleyae_Lpl2b        EPSVMVSVYGTNGDAENLQLHV-TGKIMANRTHSFLLVTEEDLGDLLMMTFKLEE-TGGW
XP_014340461.1_Lchalumnae_LPL2         EPSLFISLYGTEGEIEGLEIKV-EEKIVPNRTHSFLLVTEVFIGELLMMTFKWWE-VSEW
XP_028672354.1_Ecalabaricus_LPL2       DPTLTVSLYGTKGDAENLKLNI-KEKINPNKTHSFLLVTEVDLGELLMIQFRWEE-SASW
XP_006640053.2_Loculatus_LPL2          KPLFTVSLYGTKNDAENLHLS--TEKLSPNKTHSFLLVTEVDIGDLLMLTLRWEP-SNSW
XP_026861314.2_Eelectricus_Lpl2a       EPTITVSLYGTNGSVTNFQLDVRKGKITPNKTHSFLLVTEEDIGDLQMMKMRMED-SSRR
XP_026786600.2_Phypophthalmus_Lpl2a    ESMLTASLYGTNGNAENLHLNV-RGKIGTNTTHSFLLVTEEDIGELLMLKLKMEY-----
XP_007240187.1_Amexicanus_Lpl2a        DPTVMASLYGTNGSVENLHLKV-DGKITPNKTHSFLLVTEEDIGDLLMLKLRVED-SSNW
XP_017548831.1_Pnattereri_Lpl2a        EPTLTASLYGTSGSVENLQLKG-DDKITTNKTHSYLLVTEENIGDLLMLKLKVED-SSSW
XP_036442371.1_Cmacropomum_Lpl2a       EPTLTASLYGTSGSVENLQLKG-DDKITTNKTHSYLLVTEEDIGDLLILKLKVED-SSSW
XP_023699905.1_Pkingsleyae_Lpl2a       EPTLTVSLHGANGDAKDLHLNI-NEKIAGNNTHSFLLVTEKDIGELLMIKLKWEN-RSKW
XP_002666287.3_Drerio_Lpl2a            DPAMTISLYGTIGETEDLKLSI-QEKITSNKTHSFLVVTEKDIGDLLMLKLKMED-----
XP_042604741.1_Ccarpio_Lpl2a           EPELTVSFYGTNGKAEDLKLNI-QEKITPNKTHSFLVVTEKDIGDLLMLKFKLEE-----
XP_018583841.2_Sformosus_Lpl2a         EPTLTVSLYGTSGDTEHLQLNV-KEKISTNRTHSFLLVTEKDIGEVLMMKFKWED-SNGW
XP_019904837.1_Elucius_Lpl2a           EPSLTVYLMGTEGEVEDLKLTL-KEKIMTNKTHSFLLVAEKDIGDLLMVKFKWEE-STNW
XP_014071700.2_Ssalar                  EPSLTVSLIGTKGEVEDLKLTL-KEKITTNKTHSFLLVAEKDIGDLLMVKFKWEE-STSW
XP_021459327.2_Omykiss                 EPSLTVSLIGTKGEVEDLKLTL-KEKMTSNKTHSFLLVAEKDIGDLLMVKFKWEE-STSW
XP_045553204.1_Ssalar                  EPSLTVSLIGTKGEVEDLKLTV-KEKITTNKTHSFLLVAEKDIGDLLMVKFKWQE-STNW
XP_021435376.2_Omykiss                 EPSLTVSLIGTKGEVEDLKLTL-KEKITTNKTHSFLLVAEKDIGDLLMVKFKWQE-STNW
XP_004068405.1_Olatipes_Lpl2a          QPSLSVSLYGTKGDAEDLELRL-EERLLTNKTHSFLLVTEKDIGDLLMLKFKWEE-PSTW
XP_040040767.1_Gaculeatus_Lpl2a        EPSLTVSLHGTKGEAEDLELRL-KEKIATNKTHSFLLVTEKDIGDLLMLKFRWEE-TNGW
NP_001292532.1_Trubripes_Lpl2a         EPSLTVSLYGTKGEAENLELKL-KEKIAVNKTHSFLLVTEKDVGDLLMLTFKWEE-TNSW
XP_030228828.1_Gmorhua_Lpl2a           EPSLTVSLYGTKGDAENLELKL-KEKIATNKTHSFLLVTEQDIGDLLMLKFRWEE-DKGW
XP_008333524.1_Csemilaevis_Lpl2a       EPSLTVSLYGTKGETENLQLKL-KEKIATNKTHSFLLVTEKDIGDLLMLKFKWEE-TNSW
XP_029905846.1_Mmurdjan_Lpl2a          EPSLTVSLYGTKDEAENLELKL-KEKISTNKTHSFLLVTEKDIGDLMMLKFKWEETTSGW
XP_003439890.1_Oniloticus_Lpl2a        EPSLTVSLYGTKGEAENLQLKL-KEKIVTNKTHSFLLVTEKDIGDLLMLKFKWEE-TNGW
XP_035504157.2_Smaximus_Lpl2a          EPSLTVSLYGTKGEAENLELKL-KEKIATNRTHSFLLVTEKDIGDPLMLKFKWEE-TNSW
XP_039667734.1_Pfluviatilis_Lpl2a      EPSLTVSLYGTKGEAENLELKL-KEKIATNRTHSFLLVTEKDIGDLLMLKFKWEE-TNGW
XP_051233284.1_Dlabrax_Lpl2a           EPSLTVSLYGTKGEAENLELKL-KEKIATNKTHSFLLVTEIDIGDLLMLKFKWEE-TNGW
XP_030639366.1_Cchanos_Lpl2a           EPSLSVSLYGTNGEAENLQLNG-DERIAANKTHSFLLVTEKDLGDLLMLTFRWEE-SSSW
XP_012683807.1_Charengus_Lpl2a         DPSLTVSLHGTKGDAENLPLNV-KEKIAANRTHSFLLVAEKDIGDLLMLKFRWEE-STSW
XP_035270726.1_Aanguilla_Lpl2a         EPSLTVSLYGTKGDAEDLQLNI-KEKISTNKTLSFLLVTEKDIGELLMIKFKWEE-SSSW
XP_036374600.1_Mcyprinoides_Lpl2a      EPSLTVSLYGTKGDAEDLQLNM-KDKISVNKTHSFLLVTEKDIGDLLMMKFKWEE-SSSW
                                       .. .   .*: ..   : :     .:  * * *:*:*:*  :*:  :: :.

XP_007899210.1_Cmilii_LPL2             DSLWEQVKNSISWAKLPFWTSSVVSSSDIHVRRIRVKSGEMQKK----------------
XP_023674674.1_kingsleyae_Lpl2b        STS-SLMKMVSSW-----WSGDDANSSHIMVRKIRVKAGETQKK----------------
XP_014340461.1_Lchalumnae_LPL2         SSA-SLMQMLSSW-----WSGQS-SPAETEIHNIHVLSGETQQK----------------
XP_028672354.1_Ecalabaricus_LPL2       SSL-SLLKMFSTW-----WSGEKVPDSSVPIHRIRVKSGEAQTR----------------
XP_006640053.2_Loculatus_LPL2          SPS-SLVRAVSSW-----WSSKKPAGSSMEIRKVQVKAGESQKK----------------
XP_026861314.2_Eelectricus_Lpl2a       PVS-SLISTAWSW-----WNGDS-SCSEIKAKKISIRTGETQKKQTRCRCVQVWLQTNSP
XP_026786600.2_Phypophthalmus_Lpl2a    -SS-SLLNMMWPW-----WNGDS---PELHVRKISIWIGETQKK----------------
XP_007240187.1_Amexicanus_Lpl2a        SAS-SLFGMVQSW-----WNGES-SDSEPQIQRIRIRIGETQKK----------------
XP_017548831.1_Pnattereri_Lpl2a        SVS-SLLSKAQSW-----WNGDT-TGSEPQVKKIRIWIGETQKK----------------
XP_036442371.1_Cmacropomum_Lpl2a       SAS-SLFSKARSW-----WNGDT-TGSEPKVKKIRIRIGETQKK----------------
XP_023699905.1_Pkingsleyae_Lpl2a       TTS-FMLKMVSSW-----WPGDSTLGSDMEVHKIRVKAGETQKR----------------
XP_002666287.3_Drerio_Lpl2a            SSS-SLLNLLSSW-----WSGDP-TDSGLRVQKIRIRAGETQKK----------------
XP_042604741.1_Ccarpio_Lpl2a           STS-SLLSLLSSW-----WYTDS-ANSGIQVHKIRVRVGETQKK----------------
XP_018583841.2_Sformosus_Lpl2a         SSF-SLRKSLSAW-----WSGNSAASADIQVHKIWVKVGETQKK----------------
XP_019904837.1_Elucius_Lpl2a           SAS-SMLRMFSSW-----WSGDS-ADSEVAVHKIRIRVGETQKK----------------
XP_014071700.2_Ssalar                  SAS-FMLNMVSSW-----WSGDS-AESEVEVHKIRIRVGETQKK----------------
XP_021459327.2_Omykiss                 SPS-FMLNMVSSW-----WSGDS-AKSEVEVHKIRIRVGETQKK----------------
XP_045553204.1_Ssalar                  SAS-SMLKMVSSW-----WSGDS-ADSEVEVHKIRIRVGETQKK----------------
XP_021435376.2_Omykiss                 SAS-SMLKMVSSW-----WSGDS-ADSEVEVHKIRIRVGETQKK----------------
XP_004068405.1_Olatipes_Lpl2a          TAS-SLLKMVSSW-----WSGDS--NGGMEVHKIRIRAGETQKR----------------
XP_040040767.1_Gaculeatus_Lpl2a        SAS-NMLKMVSSW-----WSGDS-DSNDMEVHKIRIRAGETQQK----------------
NP_001292532.1_Trubripes_Lpl2a         SSS-RLLKMVSSW-----WGGDS-GGADVQVHKIRVRAGETQQK----------------
XP_030228828.1_Gmorhua_Lpl2a           SAS-SMLKMVSSW-----WSGDP-DSSDMAVHRIRVRVGETQQK----------------
XP_008333524.1_Csemilaevis_Lpl2a       SAS-SMLKMVSSW-----WSGDS-ESSDMEVHKIRIRAGESQQK----------------
XP_029905846.1_Mmurdjan_Lpl2a          SAS-NMLRMVSSW-----WSGDS-DDTGMEVHKIRIRVGETQQK----------------
XP_003439890.1_Oniloticus_Lpl2a        SAS-SMLKMVSSW-----WSGSS-DSANVTVHKIRIRAGETQQK----------------
XP_035504157.2_Smaximus_Lpl2a          SAS-SMLKMVSSW-----WSGDS-DNGNMEVHKIRIRAGETQQK----------------
XP_039667734.1_Pfluviatilis_Lpl2a      SAS-NMLKMVSSW-----WSGDS-DSGDMEVHKIRVRAGETQQK----------------
XP_051233284.1_Dlabrax_Lpl2a           SAS-NMLKMVSSW-----WSGDT-DNANMEVHKIRIRAGETQQK----------------
XP_030639366.1_Cchanos_Lpl2a           TAA-SLLKKVSSW-----WSGDS-AGADVRVQKIRMRVGETQKK----------------
XP_012683807.1_Charengus_Lpl2a         SAS-SLLRMVSSW-----WSGDS-ATPSIDVHKIRIRVGETQKK----------------
XP_035270726.1_Aanguilla_Lpl2a         STS-SLLNMVSSW-----WSSDSSANSDVEVHKIRVKVGETQKK----------------
XP_036374600.1_Mcyprinoides_Lpl2a      SSS-SLLQMVSSW-----WSGNSAATSGVEVHKIRVKVGETQKK----------------
                                                  .*     *  .         ..: :  ** * .

XP_007899210.1_Cmilii_LPL2             --------------MVFCLKDVSAAIIQSQEVIFIKCPPKVEQV-LRAPNQTIAGHY---
XP_023674674.1_kingsleyae_Lpl2b        --------------LVFCMKDTQPLNL-AKEVTFVKCEDGWKAS----SRRRRIRRD---
XP_014340461.1_Lchalumnae_LPL2         --------------MVFCPKHSSASPK-IQEVTFVRCQDYWSKS----QKRLS-RKH---
XP_028672354.1_Ecalabaricus_LPL2       --------------MVFCSKDSPEFML-DQALTFVRCQEGWKRQ----PNRPATKTH---
XP_006640053.2_Loculatus_LPL2          --------------MAFCAKDVRTLAP-SQEITFVKCQDEWNSG----SKRNTEKT----
XP_026861314.2_Eelectricus_Lpl2a       PTVHGRPNHLTQGRMMFCLKESKKASL-SPEVTFVKCT-AWGKS----CRRHNFNG----
XP_026786600.2_Phypophthalmus_Lpl2a    --------------VVFCSKDLQNTNL-LHEVIFVKCKNTWRRS----SMRRKYKSE---
XP_007240187.1_Amexicanus_Lpl2a        --------------MVFCLKFYQKANL-SHEATFVKCKESWRKS----SKRTRGRSIEDT
XP_017548831.1_Pnattereri_Lpl2a        -------------   FCLKDHQKSRT-SHEATFVKCKETWRKA----SRRAH-------
XP_036442371.1_Cmacropomum_Lpl2a       --------------LVFCLKDRQKSST-SHEATFVKCKETWRKS----SKRHTERTH---
XP_023699905.1_Pkingsleyae_Lpl2a       --------------MVFCLKNPETPGV-SQELSFVKCKESWKFH----SKRRGLRRQ---
XP_002666287.3_Drerio_Lpl2a            --------------MVLCLKDPQTPGL-AQDFVFVKCKESWRSS----SKRSKLKSH---
XP_042604741.1_Ccarpio_Lpl2a           --------------IVLCLKDPQTGSS-SQDVVFVKCKEPWRYL----SKRPKLKSQ---
XP_018583841.2_Sformosus_Lpl2a         --------------IVFCLKNPSIANL-AQEIVFVKCKDDWRNP----SKRYGPKIQ---
XP_019904837.1_Elucius_Lpl2a           --------------MVFCIKHPQDLSL-QKEVTFVKCKEEWKKT----SKRLNLGNH---
```

```
XP_014071700.2_Ssalar                 --------------MEFCIKDPHALSL-QQEVTFVKCKDEWRKT----SKRVNLGNH---
XP_021459327.2_Omykiss                --------------MVFCIKDPHALSL-QQEVTFVKCKDEWRKT----SKRVNLGKH---
XP_045553204.1_Ssalar                 --------------MVFCIEGPHALSL-QQEVTFVKCKINGKKQ----T-----------
XP_021435376.2_Omykiss                --------------MLFCIKGPHALSL-QQEVTFVKCKINRKKQ----T-----------
XP_004068405.1_Olatipes_Lpl2a         --------------MVFCVKEPQGF---SQEVTFVKCKDAWRMN----RKKTERGTVEIR
XP_040040767.1_Gaculeatus_Lpl2a       --------------MVFCVKDNEAQKF-QQEVTFVKCKGAWRRN-----QRRTLKRK---
NP_001292532.1_Trubripes_Lpl2a        --------------MVFCVKNPDVQNL-MREVTFVRCKDAWRTS----GKRVTLENHRP-
XP_030228828.1_Gmorhua_Lpl2a          --------------LVLCVKDPEALSL-TQEITFVKCRADWRKT---GANRGTPK-----
XP_008333524.1_Csemilaevis_Lpl2a      --------------LVFCVKDPEVHTL-TQEITFVRCQDSWKKN----PKRVSLEKH---
XP_029905846.1_Mmurdjan_Lpl2a         --------------VVFCVKDPQTQSL-AQEVTFVKCKGEWRTNSKRTPKRITLENH---
XP_003439890.1_Oniloticus_Lpl2a       --------------MVFCIKDPDAQTL-TQELTFVKCKDPWRTNRKRTPRRVTLENH---
XP_035504157.2_Smaximus_Lpl2a         --------------MVLCVKDPGAQSS-TQEVTFVKCKDTWRTNSKQTPKRITLENR---
XP_039667734.1_Pfluviatilis_Lpl2a     --------------MVFCVKDPEAQKL-TQEVTFVKCKDAWRTNLTSTPKRVTLENH---
XP_051233284.1_Dlabrax_Lpl2a          --------------MVFCVKDPEAHKL-TDEVTFVKCKDLWRRNSKRTPKRITLTTENH-
XP_030639366.1_Cchanos_Lpl2a          --------------MVFCLKDPETASL-AQEVTFVKCKDDWRRL----SKR---------
XP_012683807.1_Charengus_Lpl2a        --------------IVLCVKDPKAVNM-AQEVTFVKCKDEWRSP----SRRKNLQTHSHS
XP_035270726.1_Aanguilla_Lpl2a        --------------TVFCIKSPQALNL-AQEVTFVKCMDEWKSP----SKRHVPKKH---
XP_036374600.1_Mcyprinoides_Lpl2a     --------------MVFCVKSPQALNF-AQEVTFVKCMDDWKSH----SKRHNLKRH---
                                                      :* :             *:.*

XP_007899210.1_Cmilii_LPL2            ------
XP_023674674.1_kingsleyae_Lpl2b       ------
XP_014340461.1_Lchalumnae_LPL2        ------
XP_028672354.1_Ecalabaricus_LPL2      ------
XP_006640053.2_Loculatus_LPL2         ------
XP_026861314.2_Eelectricus_Lpl2a      ------
XP_026786600.2_Phypophthalmus_Lpl2a   ------
XP_007240187.1_Amexicanus_Lpl2a       EVPIYR
XP_017548831.1_Pnattereri_Lpl2a       ------
XP_036442371.1_Cmacropomum_Lpl2a      ------
XP_023699905.1_Pkingsleyae_Lpl2a      ------
XP_002666287.3_Drerio_Lpl2a           ------
XP_042604741.1_Ccarpio_Lpl2a          ------
XP_018583841.2_Sformosus_Lpl2a        ------
XP_019904837.1_Elucius_Lpl2a          ------
XP_014071700.2_Ssalar                 ------
XP_021459327.2_Omykiss                ------
XP_045553204.1_Ssalar                 ------
XP_021435376.2_Omykiss                ------
XP_004068405.1_Olatipes_Lpl2a         SP----
XP_040040767.1_Gaculeatus_Lpl2a       ------
NP_001292532.1_Trubripes_Lpl2a        ------
XP_030228828.1_Gmorhua_Lpl2a          ------
XP_008333524.1_Csemilaevis_Lpl2a      ------
XP_029905846.1_Mmurdjan_Lpl2a         ------
XP_003439890.1_Oniloticus_Lpl2a       ------
XP_035504157.2_Smaximus_Lpl2a         ------
XP_039667734.1_Pfluviatilis_Lpl2a     ------
XP_051233284.1_Dlabrax_Lpl2a          ------
XP_030639366.1_Cchanos_Lpl2a          ------
XP_012683807.1_Charengus_Lpl2a        S-----
XP_035270726.1_Aanguilla_Lpl2a        ------
XP_036374600.1_Mcyprinoides_Lpl2a     ------
```
